# Mechanistic Studies of Alkyl Chloride Acetoxylation by Pt–Sb Complexes

**DOI:** 10.1021/acs.organomet.4c00399

**Published:** 2025-02-20

**Authors:** Christopher K. Webber, Jugal Kumawat, Fanji Kong, Diane A. Dickie, Daniel H. Ess, T. Brent Gunnoe

**Affiliations:** † Department of Chemistry, University of Virginia; Charlottesville, Virginia 22904, United States; ‡ Department of Chemistry and Biochemistry, Brigham Young University; Provo, Utah 84604, United States

## Abstract

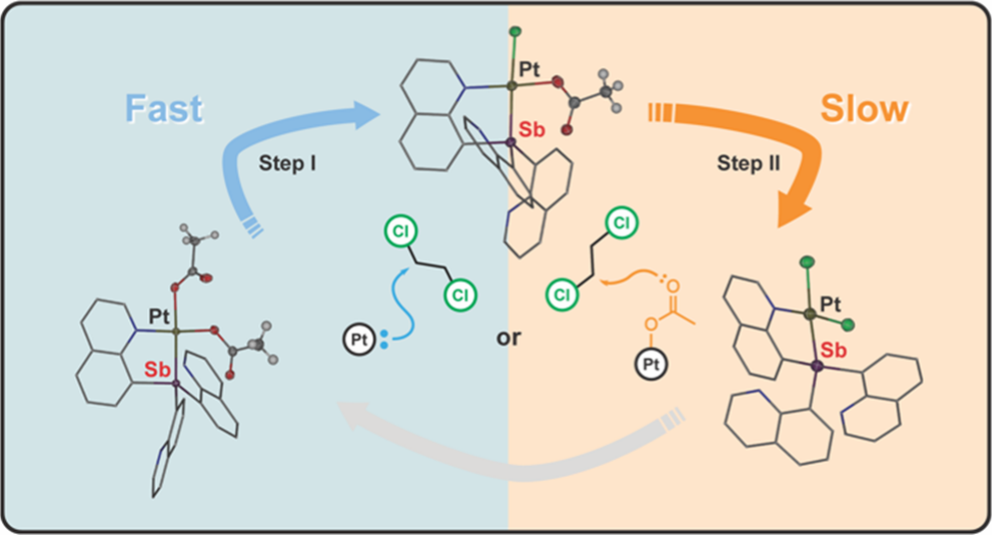

The bis-acetate complexes (SbQ_3_)Pt(OAc)_2_ (**1**) and (SbQ_2_Ph)Pt(OAc)_2_ (**2**) (Q = 8-quinolinyl) were used to study C–Cl acetoxylation of 1,2-dichloroethane (DCE) to generate 2-chloroethyl acetate and the complexes (SbQ_3_)PtCl_2_ (**1b**) and (SbQ_2_Ph)PtCl_2_ (**2b**), respectively. The first acetoxylation step produced the intermediates (SbQ_3_)Pt(Cl)(OAc) (**1a**) and (SbQ_2_Ph)Pt(Cl)(OAc) (**2a**). The reaction was studied using pseudo first order kinetics (excess DCE) in order to compare the rates of reaction of **1** and **2**, which revealed that *k*_obs_ = 2.44(6) × 10^–4^ s^–1^ for **1** and 0.51(2) × 10^–4^ s^–1^ for **2**. The intermediate **1a** was synthesized independently, and the solid-state structure was determined using single crystal X-ray diffraction. A non-Sb containing control complex, (^t^bpy)Pt(OAc)_2_ (**3**) (^t^bpy = 4,4′-di-*tert*-butyl-2,2′bipyridine), was studied for the acetoxylation of DCE to form (^t^bpy)Pt(Cl)(OAc) with *k*_obs_ = 0.46(1) × 10^–4^ s^–1^. Density Functional Theory (DFT) calculations were used to examine possible Pt-mediated mechanisms for the reactions of **1**, **2**, or **3** with DCE. The lowest energy calculated substitution mechanism occurs with nucleophilic attack by the Pt center on the C−Cl bond followed acetate reaction with the Pt−C bond. However, close in energy and potentially also a viable mechanism is a direct substitution mechanism where the coordinated acetate anion directly reacts with the C−Cl bond of DCE. In addition, the rate of acetoxylation for complex **1** in heated dichloromethane-*d*_2_ and chloroform-*d* was determined (0.43(1) × 10^–4^ s^–1^ for dichloromethane-*d*_2_ and 0.37(1) × 10^–4^ s^–1^ for chloroform-*d*) and compared to the rate of acetoxylation of DCE.

## Introduction

Multifunctional transition metal complexes containing Sb offer potential electronic flexibility controlled by various accessible Sb oxidation states.^1−4^ Sb^III^ typically coordinates to a transition metal as a σ-donor whereas Sb^V^ has an empty orbital and can function as a σ-acceptor. Therefore, depending on the Sb oxidation state, Sb can act as either a net electron-donor or an electron-acceptor.^1^

**Scheme 1:**
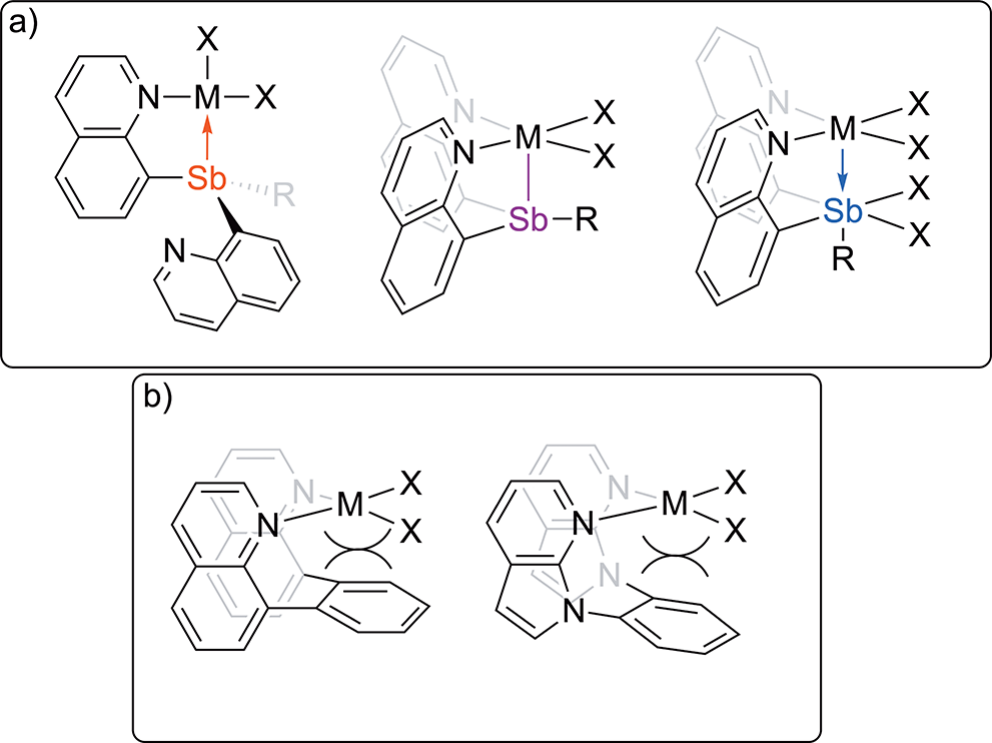
Depiction of (a) Examples of Three Binding Modes Accessible to Pt–Sb Complexes and (b) Illustration of the Role Capping Arene Ligands might Play in Blocking a Bonding Orbital

Recently, we reported quinoline based Pt–Sb complexes that differ in their formal oxidation state, and related Rh–Sb Z-type complexes were recently reported by the Gabbaï group.^5,6^ The bonding interaction between Pt and Sb is dependent on the oxidation state of both Pt and Sb (Scheme 1a). We have proposed that our newly reported Pt–Sb complexes bear some potential conceptual similarities to previously reported complexes with “capping arene” ligands^7−9^ that have been suggested to destabilize high oxidation state late transition metal intermediates by blocking preferred octahedral geometries (Scheme 1b).^10^ For example, capping arene ligands have been demonstrated in Rh catalyzed alkene hydrogenation^11^ and arene alkenylation^12^ as well as Co catalyzed water oxidation.^13^ Similar to our demonstrations with capping arene ligands, for which metal/arene bonding impacts access to and stability of reaction intermediates, access to various binding motifs between Pt and Sb and the variable oxidation states accessible to Sb can modulate the energy, and hence access, to reaction intermediates.

**Scheme 2:**
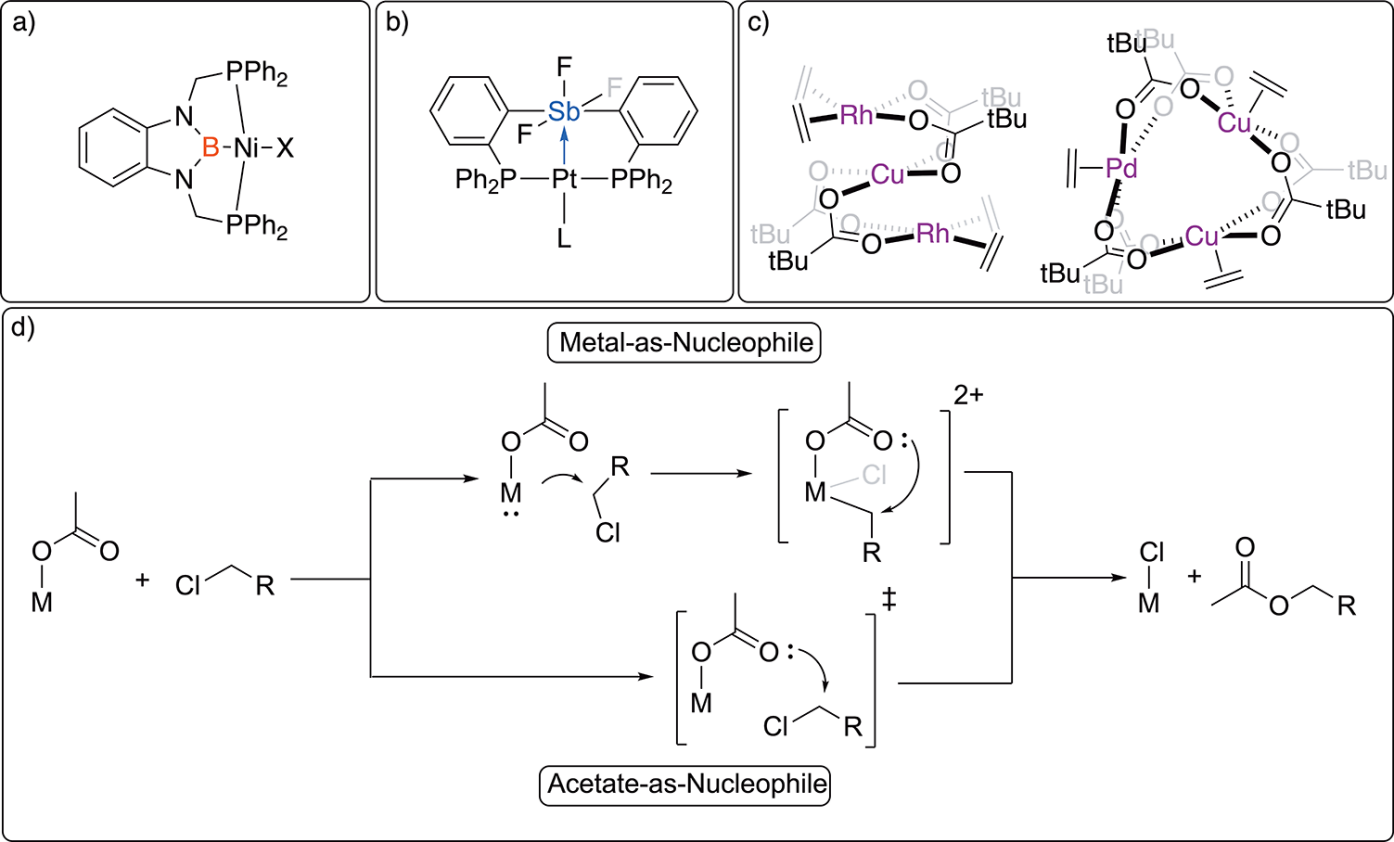
(a) Selected Examples of Multinuclear Complexes that Bear Lewis Acidic Binding Sites for Substrate Docking.^30,31^ (b) Example of Previously Reported Pt–Sb Electrophilic Catalyst Containing a Z-type Tnteraction between Pt and Sb.^33^ (c) Multi-metallic Carboxylate Complexes Reported for Oxidative Arene Alkenylation.^27,42^ (d) Two Potential Mechanisms for the Acetoxylation of C–Cl Bonds through Either Innersphere (Involving Direct Metal Involvement) Metal-as-nucleophile or Outersphere (No Direct Metal Involvement) Acetate-as-nucleophile Mechanisms

Multifunctional organometallic complexes (i.e., complexes with more than one site for substrate activation) can provide access to unique reactivity and mechanisms for small molecule activation and functionalization.^14−29^ For example, in some organometallic complexes appended Lewis acidic centers have been demonstrated to position an incoming substrate to provide enhanced reactivity. As an example of this effect, Lewis acidic boron has been demonstrated to impact the coordination of substrates prior to activation by a pendent transition metal (Scheme 2a).^30−32^ Z-type ligands, which accept donation of an electron-pair from a metal, can enhance transition metal based electrophilic reactivity (Scheme 2b).^33−41^ Evidence that Ir, Rh, and Pd complexes likely incorporate Cu carboxylate moieties into multimetallic complexes that are active catalysts for oxidative arene alkenylation has been reported (Scheme 2c).^24,27,42,43^ In the case of Rh, Density Functional Theory (DFT) calculations showed that the addition of Cu^II^ likely lowers the energy for benzene coordination as well as the activation barrier for O–H reductive coupling.^27^ For Pd, the incorporation of Cu^II^ appears to force geometric strain that benefits the complex’s ability to activate C–H bonds.^42^

The acetoxylation of various substrates by carboxylate containing metal complexes has been reported.^24,44^ The acetoxylation of alkyl halocarbons is a route to protected esters, and ester functionality can be cleaved to provide access to alcohols.^45^ Previously, AgOAc has been demonstrated to perform the acetoxylation of halocarbons at elevated temperatures.^46^ NaOAc and KOAc in the presence of crown ether ligands can perform the acetoxylation of halocarbons.^47^ Given the presence of Pt and OAc in our Pt–Sb complexes, we envisioned that either could function as nucleophiles through metal-as-nucleophile (i.e., innersphere) or acetate-as-nucleophile (i.e., outersphere) mechanism (Scheme 2d).

Herein, we sought to further explore Pt–Sb carboxylate complexes by reacting them with substrates that contain C–Cl bonds to better understand the role (if any) that the Sb center plays in C–Cl acetoxylation. Given our previous work with oxidized Pt–Sb complexes,^6^ we envisioned that the acetoxylation of alkyl chlorides offer an opportunity to understand: (a) if the reaction occurs through an oxidative addition type mechanism (metal-as-nucleophile), which could access different bonding interactions between Pt and Sb, or (b) if the reaction occurs through a direct nucleophilic attack by acetate (acetate-as-nucleophile), which could be affected by relative donor ability of the ligands.

We found that Pt–Sb carboxylate complexes react at elevated temperatures with the C–Cl bond of 1,2-dichloroethane (DCE) to generate the corresponding 2-chloroethyl acetate acetoxylation product and Pt–Sb bis-chloride complexes. Experiments and DFT calculations have been used to examine the role of the Pt and Sb centers as well as the quinoline ligand. A control Pt-acetate complex containing a bipyridine ligand was synthesized and reacted with DCE to provide a comparison with a non-Sb containing ligand. Through kinetic studies of the first step of the acetoxylation reaction, we demonstrated that the reaction rate changes as a function of the Sb ligand’s arene substituents. Furthermore, DFT calculations indicated two viable mechanisms for which the Pt or acetate ligand may initiate nucleophilic addition to DCE to generate the acetoxylated products. Subtle electronic differences between the two Sb ligands appear to be the primary cause of the observed ligand effect as opposed to direct involvement of quinoline coordination or unique Sb binding interactions. Also, the acetoxylation reactions of CD_2_Cl_2_ and CDCl_3_ were studied providing a comparison with various alkyl chloride substrates.

## Results and Discussion

### Experimental Analysis of Reactions with DCE

**Figure 1:**
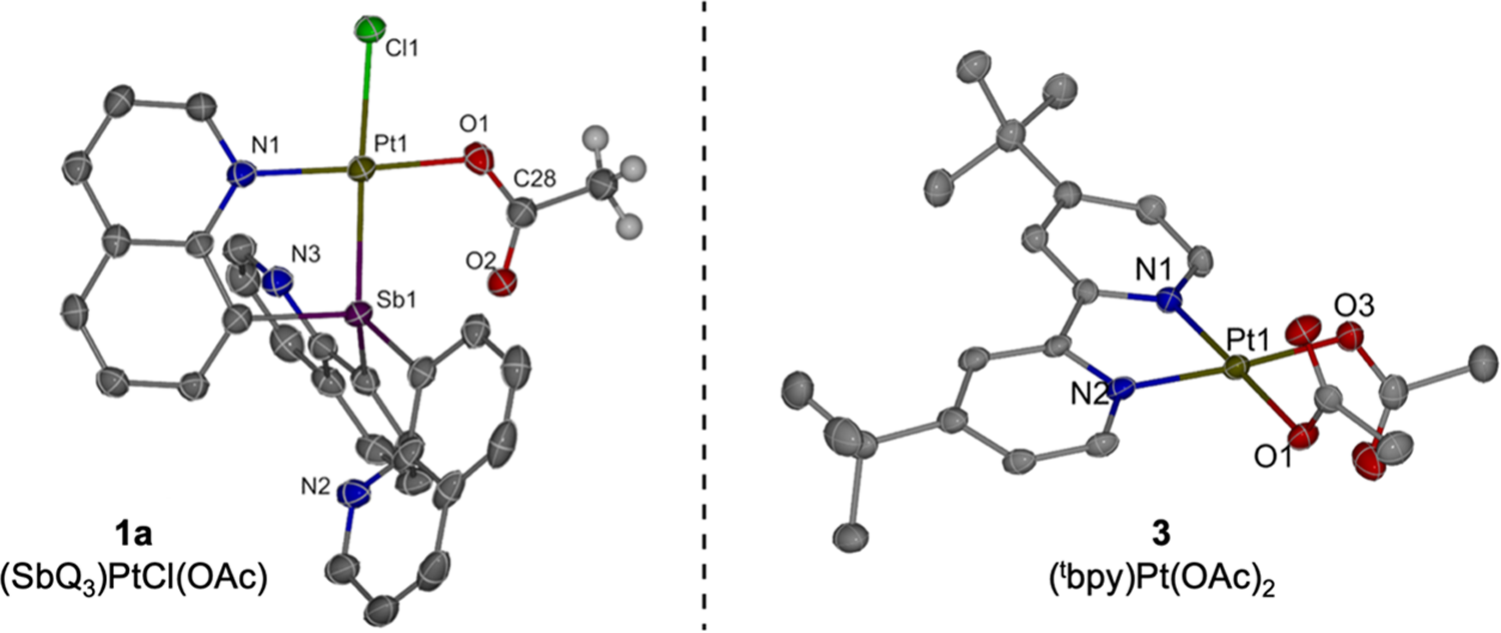
ORTEPs of (SbQ_3_)Pt(Cl)(OAc) (**1a**) and (^t^bpy)Pt(OAc)_2_ (**3**). Ellipsoids are drawn at the 50% probability level and some hydrogen atoms and noncoordinating solvents are omitted for clarity. Selected bond distances for **1a** (Å): Pt1–Sb1 2.4558(4); Pt1–Cl1 2.3871(12); Pt1–O1 2.017(3); O1–C28 1.277(6); O2–C28 1.227(6); Sb1···O2 2.619(3); H_a_···Cl1 2.469. Selected bond distances for **3** (Å): Pt1–N1 1.986(3); Pt1–N2 1.994(3); Pt1–O1 2.022(3); Pt1–O2 2.016(3). (Two molecules of **3** are present in asymmetric unit. The bond lengths of one molecule are included here).

**Scheme 3:**
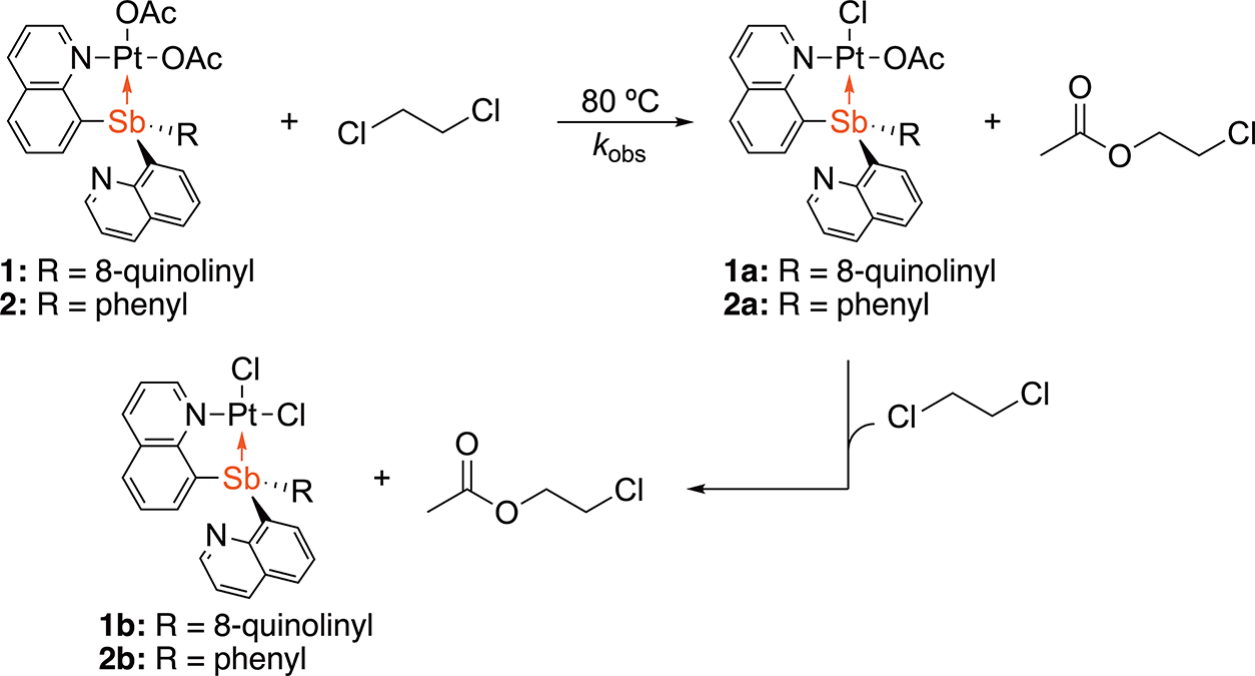
Acetoxylation of DCE by (SbQ_3_)Pt(OAc)_2_ (**1**) and (SbQ_2_Ph)Pt(OAc)_2_ (**2**) Resulting in the Formation of 2-Chloroethyl Acetate and (SbQ_3_)Pt(Cl)(OAc) (**1a**) and (SbQ_2_Ph)Pt(Cl)(OAc) (**2a**), Respectively

The complexes (SbQ_3_)Pt(OAc)_2_ (Q = 8-quinolinyl) (**1**) and (SbQ_2_Ph)Pt(OAc)_2_ (**2**) were found to mediate the acetoxylation of 1,2-dichloroethane to form 2-chloroethyl acetate with concomitant generation of chloride complexes (SbQ_3_)PtCl_2_ (**1b**) and (SbQ_2_Ph)PtCl_2_ (**2b**) (Scheme 3) upon heating at 80 °C in neat DCE (utilizing a sealed capillary containing CDCl_3_ as the locking solvent). Upon closer investigation of the reaction by in situ ^1^H NMR spectroscopy, a partial chloride abstraction product, (SbQ_3_)Pt(Cl)(OAc) (**1a**), was identified as an intermediate. Previous investigations of complexes **1b** and **2b** indicated a downfield shifted resonance in the ^1^H NMR spectrum at >11 ppm, which is likely due to a through space interaction with the chloride anion coordinated to Pt.^6^ This proton resonance shifted upfield to ∼10 ppm for **1** and **2** due to the presence of an acetate instead of a chloride anion at this position. Complex **1a** was synthesized in bulk by dissolving **1** in DCE and heating to 100 °C to furnish the monochloride complex **1a**. The solid-state structure of **1a** shows the chloride trans to the Sb center (Figure 1, left), which is consistent with the observation of a downfield-shifted proton at 11.2 ppm (assigned to the proton ortho to the nitrogen in the coordinated quinoline) in ^1^H NMR spectrum of **1a**.^6^ Likewise, the >11 ppm chemical shift for one H atom of complex **2a** suggests a similar structure to **1a**. The position of the chloride ligand trans to Sb can be attributed to (a) the stronger trans effect of Sb compared to quinoline and/or, (b) the formation of a potential acetate bridging product that directs the location of OAc with the C=O pointing toward the Lewis acidic Sb center. Given the relatively close through space distance between Sb1···O2 {2.619(3)} for **1a** in the solid state, we considered that a bridging type motif might exist in solution. Previously we performed EXSY NMR spectroscopy of **1** and **2** and demonstrated that the acetate ligands do not exchange on the NMR time scale. This provides evidence of different acetate binding interactions in solution but does not conclusively show that a bridging motif persists in solution.^6^ Therefore, the acetoxylation of DCE is likely achieved through both bis-acetate (*i.e*., **1** or **2**) and monoacetate complexes (*i.e*., **1a** or **2a**) in a tandem fashion.

**Figure 2:**
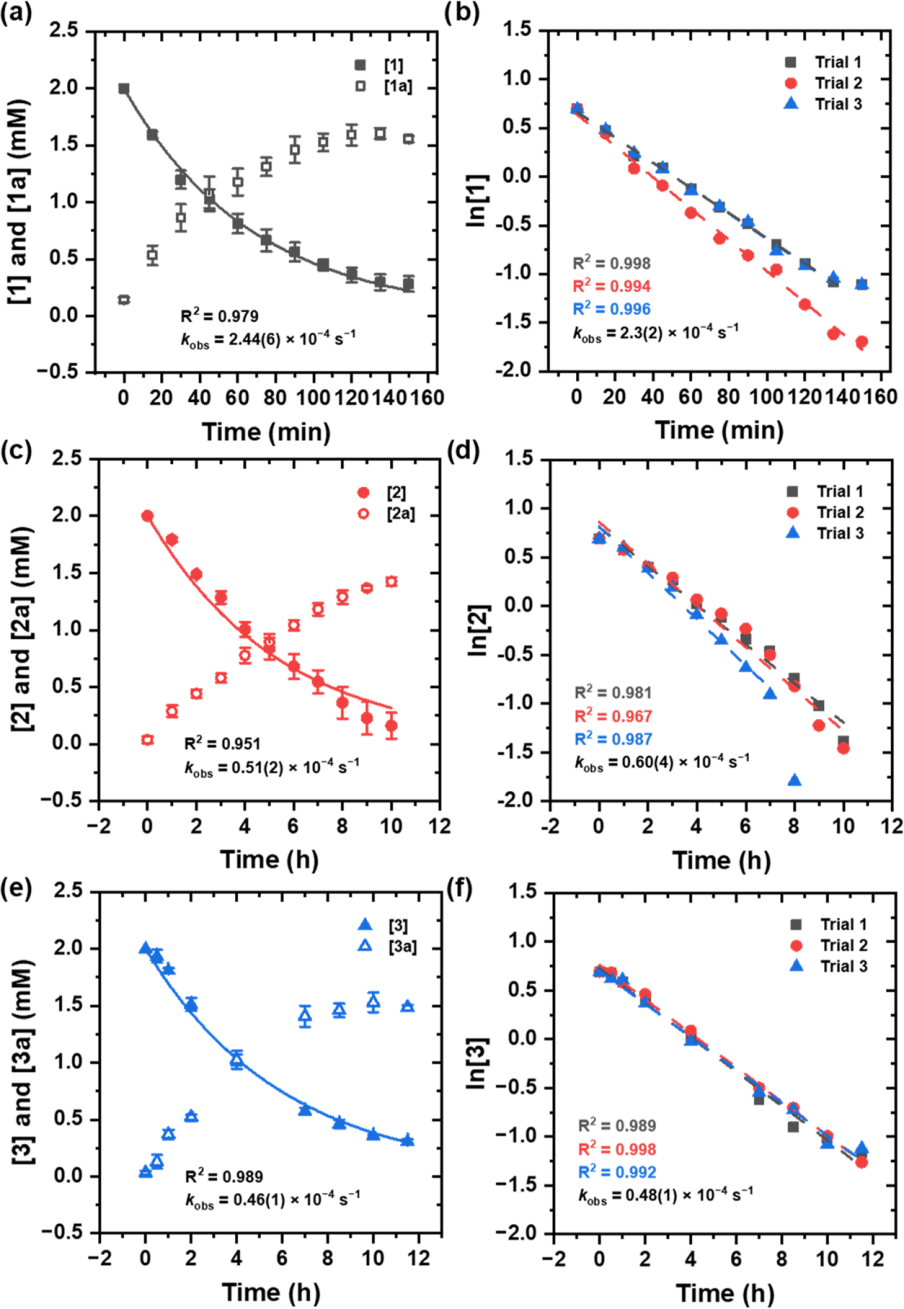
Kinetic studies of the acetoxylation of DCE using complex **1**, **2**, or **3** at 80 °C. (a) Plot of concentrations of complexes **1** and **1a** versus time. (b) Plot of ln[**1**] versus time. (c) Plot of concentrations of complexes **2** and **2a** versus time. (d) Plot of ln[**2**] versus time. (e) Plot of concentrations of complexes **3** and **3a** versus time. (f) Plot of ln[**3**] versus time. Rate = *k*_1_[**2**][DCE]^X^ = *k*_obs_[**2**], for which *k*_obs_ = *k*_1_[DCE]^X^ (superscript X denotes that the order in [DCE] is not known). The observed rate constant was calculated via a first-order exponential fit and through linearization (the *k*_obs_ calculated via a first-order exponential fit is shown in the main text).

The formation of bis-chloride complexes **1b** or **2b** from the reaction of complexes **1** or **2** with DCE could not be monitored accurately using ^1^H NMR spectroscopy due to their poor solubility under reaction conditions. Thus, the kinetics of acetoxylation of DCE at 80 °C was quantified by monitoring the disappearance of **1** or **2** and formation of **1a** or **2a**. Under pseudo-first order conditions (*i.e*., excess DCE), the reaction was found to be first order in the concentration of Pt complex, and the observed first-order reaction rate constant (*k*_obs_) was found (Figure 2, and Supporting Information Section 1). Time points near the end of the reaction deviate slightly from linearity, which is likely due to either the formation of bis-chloride products **1b** and **2b** or low signal-to-noise ratio of mostly consumed **1** and **2** (see Supporting Information for more details).

The Pt(II) complex (^t^bpy)Pt(OAc)_2_ (**3**) (^t^bpy = 4,4′-di-*tert*-butyl-2,2′-bipyridine) was synthesized to provide a non-Sb containing Pt bis-acetate complex to compare rates of acetoxylation. Heating PtCl_2_ and ^t^bpy in MeCN at 85 °C furnished (^t^bpy)PtCl_2_ as a yellow powder (the ^1^H NMR spectrum was consistent with reported spectra in CDCl_3_).^48^ AgOAc was then added to solutions of (^t^bpy)PtCl_2_ in DCM to form **3** as a brown powder. Complex **3** was characterized by ^1^H and ^13^C{^1^H} NMR spectra as well as single crystal X-ray diffraction (Figure 1, right). Upon heating **3** in DCE at 80 °C, the monochloride product (^t^bpy)Pt(Cl)(OAc) (**3a**) was observed. The kinetics of the disappearance of **3** and appearance of **3a** was monitored by ^1^H NMR spectroscopy in the same manner as **1** and **2** to provide a non-Sb containing Pt bis-acetate complex comparison.

Based on the initial tests, the acetoxylation of DCE using complex **1** reached completion within 3 h. Therefore, the reaction was monitored for 150 min total using 15 min intervals. The yield determined by ^1^H NMR spectroscopy was 73(4)% of **1a** with 19(4)% of **1** remaining. In contrast, the reaction with complex **2** requires longer reaction times. Thus, we monitored the reaction of **2** with DCE for 10 h using 1-h intervals. The yield determined by ^1^H NMR spectroscopy was 75(7)% of **2a** with 10(2)% of **2** remaining. A clear ligand effect was observed for the acetoxylation of DCE using complex **1** vs complex **2**. The observed rate constant was calculated via a first-order exponential fit and through linearization. The observed rate constant (calculated via first-order exponential fitting) for the reaction using complex **1** is approximately 5-fold that of complex **2** (*k*_obs_ = 2.44(6) × 10^–4^ s^–1^ for **1** vs 0.51(2) × 10^–4^ s^–1^ for **2**). The difference in rate between complexes **1** and **2** is likely due to difference between a quinoline vs phenyl substituent on the Sb which could either alter electron density at Sb or allow an additional nitrogen coordination. The acetoxylation of DCE with complex **3**, which did not contain Sb, was monitored for 11.5 h and had an observed rate constant (calculated via first-order exponential fitting) of 0.46(1) × 10^–4^ s^–1^, which is slower than **1** and similar to the rate of acetoxylation by **2**. The yield of **3a** by ^1^H NMR spectroscopy was 74(1)% with 15(1)% of **3** remaining. This prompted us to use DFT calculations to examine plausible reaction mechanisms and establish the reason for the differences in reactivity between complexes **1**, **2**, and **3**.

### Computational Modeling of Reaction Pathways

The reaction of complex **1** or **2** with DCE generates the respective dichloride complex **1b** or **2b** and 2-chloroethyl acetate. Our B2-PLYP//M06 DFT calculations (see Experimental Section and Supporting Information for additional details) modeled the complete Pt–Sb complexes, and structures and energies were evaluated using the CPCM continuum model, which provides an estimate of general bulk-like solvent stabilization. For metal acetate promoted acetoxylation reactions a variety of reaction mechanisms have been proposed ranging from polar mechanisms to open-shell radical reaction pathways.^49^ These mechanisms are substrate and metal complex dependent. For example, acetoxylation of aldehydes and ketones has been reported using Pb(OAc)_4_^50,51^ while acetoxylation of allylic substrates often is proposed to occur through allylic η^3^ coordination intermediates, especially for Pd complexes.^52,53^

**Scheme 4:**
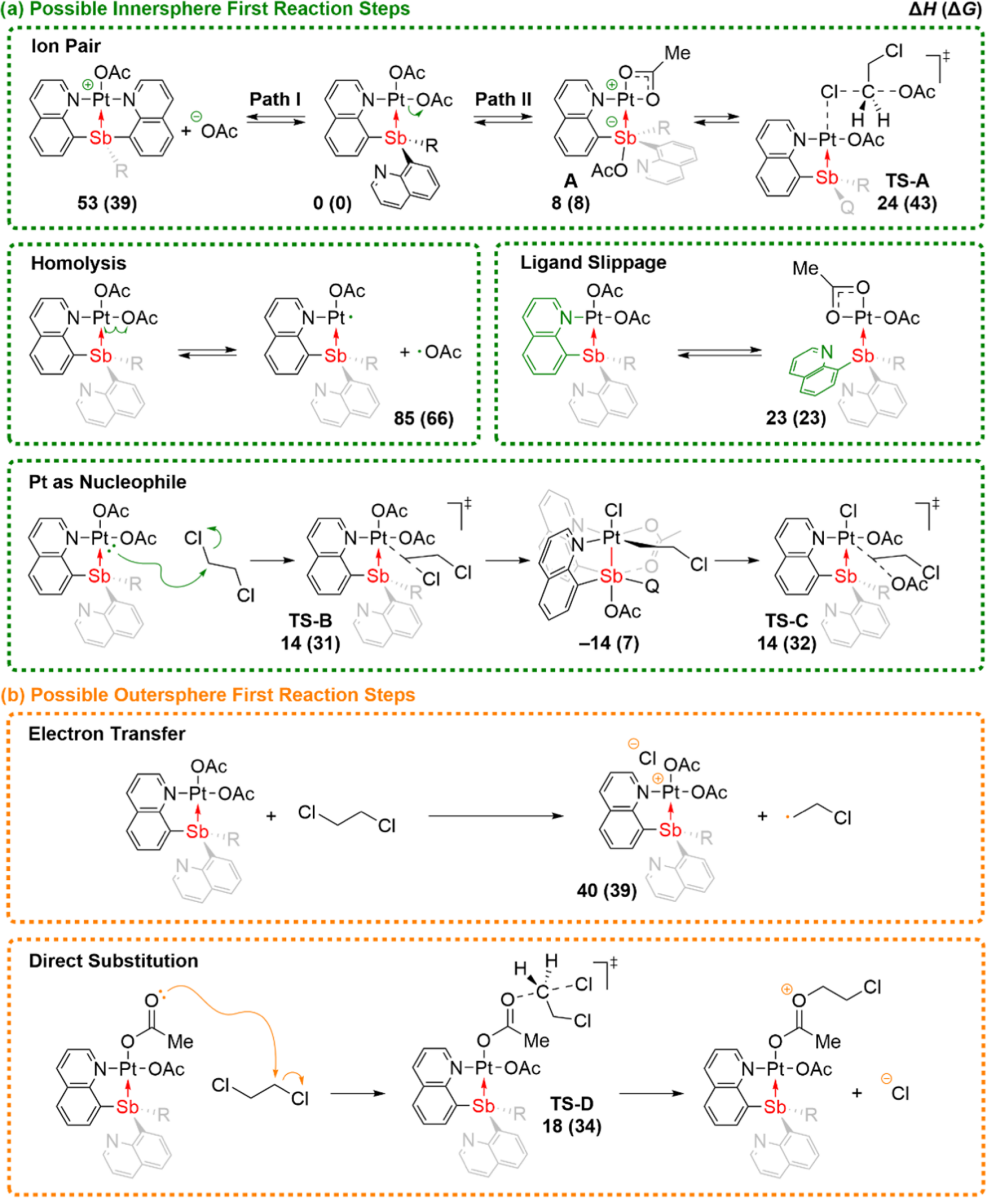
General Mechanisms for the First Reaction Step of Acetoxylation Examined with DFT Calculations B2-PLYP/def2-TZVPD//M06/def2-SVP calculated enthalpies and free energies (enthalpies are shown first, and free energies are shown in parentheses) relative to (SbQ_3_)Pt(OAc)_2_ (**1**) and DCE (kcal·mol^–1^) are shown. *Note:* Q = 8-quinolinyl. R = 8-quinolinyl for the energies provided. Enthalpies and free energies were calculated at 100 °C.

We examined the possibility of polar, radical, innersphere, and outersphere type reaction mechanisms. Scheme 4 outlines several possible first reaction steps. We did not consider disproportionation steps since the kinetic experiments showed a first-order dependence on the Pt complexes. Scheme 4a depicts innersphere mechanisms that use the Pt metal center by formation of a vacant or partially vacant coordination site at Pt through either Pt–OAc bond heterolysis/ion pair formation, Pt–OAc bond homolysis, or quinoline ligand slippage. Ion pair formation has the possibility of a variety of coordination modes and a continuum from a tight ion pair to a solvent separated ion pair. Also, Scheme 4a shows the possibility that the Pt center can directly act as a nucleophile, and this would formally be the first step of oxidative addition. Scheme 4b shows possible first reaction steps for outersphere type mechanisms with the possibility of Pt-to-DCE electron transfer or an acetate ligand acting as a nucleophile toward DCE while remaining coordinated to the Pt center.

As might be anticipated from a reaction in nonpolar solvent, acetate dissociation from complex **1** to generate a solvent separated ion pair is unlikely with a calculated enthalpy change of 53 kcal·mol^–1^, even with an acetate ligand undergoing κ^1^ to κ^2^ coordination change and a second quinoline ligand providing *N*-coordination to the Pt center. Therefore, we examined a variety of tight ion pairs for which the acetate either remains close to the quinoline ligand, bridges, or is transferred to the Sb center. The lowest energy coordination configuration identified was acetate migration to close contact with the Sb center and the remaining acetate converting to κ^2^ coordination at the Pt center. The enthalpy and Gibbs energy of this tight ion pair, **A**, is 8 kcal·mol^–1^ relative to starting complex **1**. From **A** this ion pair pathway involves a transition state for acetate to induce substitution of DCE for which the new C–OAc bond is formed simultaneous to capture of the chloride by Pt. This transition state is a backside S_N_2 style structure with the chloride being captured by the Pt center. To achieve this transition state, DCE must coordinate to the Pt center through the chlorine and the acetate must dissociate from Sb and perform the required S_N_2 attack on the backside of the Pt–Cl adduct. The subsequent substitution transition state **TS-A** has a Δ*H*^‡^ value of 24 kcal·mol^–1^ with Δ*G*^‡^ = 43 kcal·mol^–1^ and an overall reaction energy change of −11 kcal·mol^–1^ to give **1a**. While there is an energy penalty for acetate dissociation, **TS-A** is then enabled by the DCE coordination and capture of the chloride leaving group at the Pt center.

Homolysis of the Pt–OAc bond is unlikely with a calculated bond enthalpy of 85 kcal·mol^–1^ (Gibbs bond energy is calculated to be 66 kcal·mol^–1^) to generate a pair of radicals. Also, quinoline ligand slippage for which the quinolinyl arm dissociates from the Pt metal center requires an enthalpy change of 23 kcal mol^–1^. While this pathway cannot be ruled out based on this energy alone, any pathway emanating from this process would likely have a relatively high barrier given that additional steps are required after quinolinyl arm dissociation.

Alternative to a ligand dissociation reaction step, we examined the possibility that the Pt center of complex **1** can initiate nucleophilic attack of the C–Cl bond of DCE through **TS-B**. This transition state generates a cationic Pt-alkyl intermediate with a tight ion pair to chloride, and if chloride coordinates to Pt this would be the oxidative addition intermediate. The calculated activation enthalpy (Δ*H*^‡^) for this transition state is only 14 kcal·mol^–1^. Including the entropy correction, which likely would be an overestimate in this type of calculation, the Gibbs activation energy (Δ*G*^‡^) is 31 kcal·mol^–1^ relative to **1** and DCE. With a basis that contains diffuse functions to model the developing anions both B2-PLYP and M06 functionals show **TS-B** to be several kcal·mol^–1^ lower than **TS-A** (see the Supporting Information since basis sets without diffuse functions give a qualitatively different result). Perhaps surprisingly, B2-PLYP/def2-TZVPD predicts that **TS-B** is approximately 10 kcal·mol^–1^ lower in enthalpy than **TS-A**. Also, formation of the Pt^IV^ oxidative addition intermediate, which resembles the structure of a previously identified oxidized Pt–Sb complex,^6^ is exothermic. However, this intermediate is unlikely to be observable since it is endergonic (+7 kcal/mol) on the Gibbs energy surface. The subsequent transition state that leads to the substitution product through **TS-C** requires first the exchange of the acetate group for chloride followed by nucleophilic attack of the newly formed Pt–C, which would result in formation of complex **1a**. Although the oxidized intermediate in this mechanism resembles previously observed coordinatively flexible Pt–Sb quinoline structures, the impact of quinoline coordination appears to have minimal effect on the overall acetoxylation transition states.

**Figure 3:**
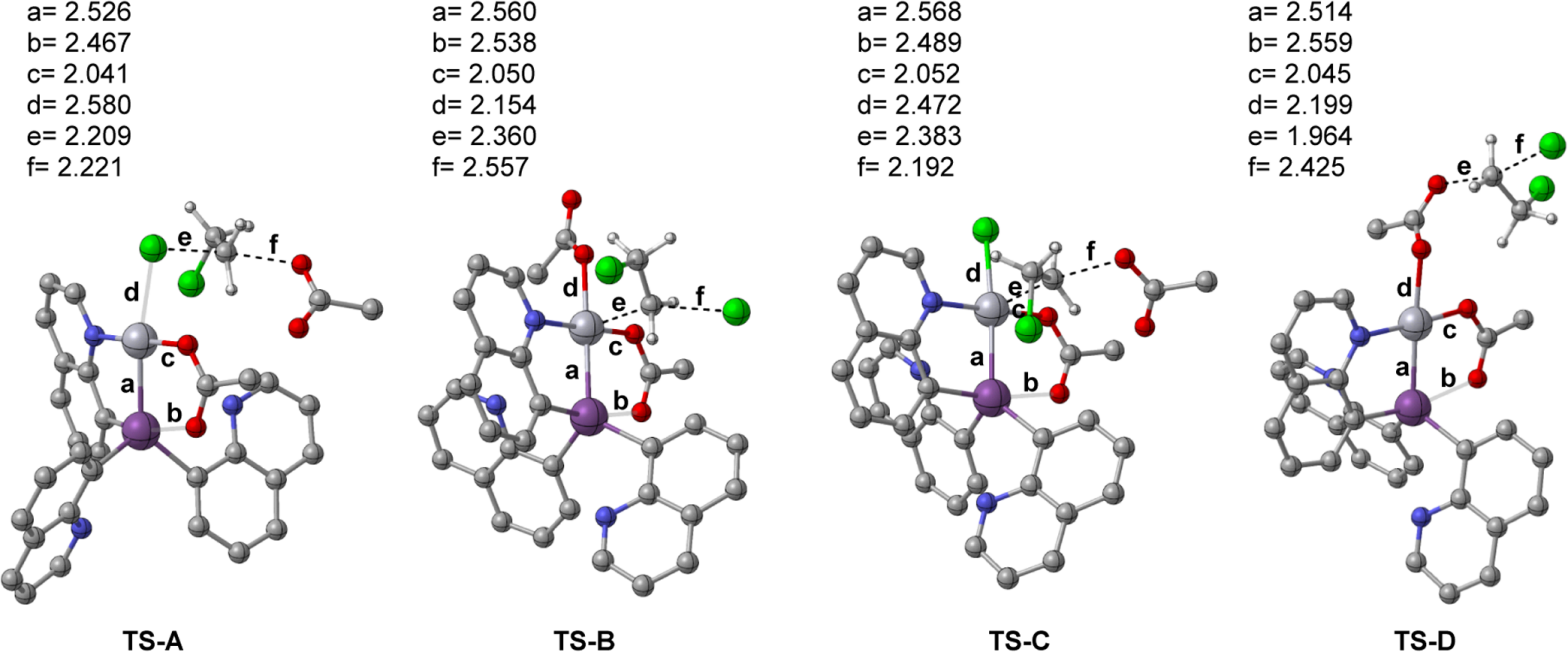
Depictions of competitive transition states, **TS-A**, **TS-B**, **TS-C**, and **TS-D**. Color coding: gray = carbon; blue = nitrogen; red = oxygen; green = chlorine; purple = antimony; dark gray = platinum; white = hydrogen. All the hydrogen atoms have been omitted except for attached to DCE. Bond distances are given in Å.

For outersphere mechanisms, we began by examining electron transfer. Initially, we considered that this could be viable with the possibility that Sb could stabilize an oxidized Pt center and the electron transfer would be coupled with DCE fragmentation to generate a carbon radical and chloride anion. Even with modeling this electron transfer process as generating a carbon radical and a tight ion pair this reaction step is highly endothermic with a calculated energy of 40 kcal·mol^–1^ and is not expected to be viable. The most viable outersphere type mechanism is the possibility that the OAc ligand acts as a nucleophile while remaining coordinated to the Pt center, which occurs through **TS-D**. This type of pathway is potentially advantageous because there is no energy penalty for making an initial ion pair intermediate or oxidizing the Pt center. The Δ*H*^‡^ for this direct substitution reaction pathway by **TS-D** is 18 kcal·mol^–1^ with Δ*G*^‡^ = 34 kcal·mol^–1^. Perhaps because there is so little electronic and geometric change required to achieve **TS-D**, this transition state is close in energy to the **TS-B** and **TS-C**. While the enthalpy and Gibbs free energy barriers for **TS-D** are higher than **TS-B** and **TS-C**, the energy difference is likely too close for DFT calculations to be definitive about the operating pathway, especially since these are both highly polarized transitions states and we used a continuum solvent model (Figure 3 shows depictions of transition states **TS-A** through **TS-D**).

With the change from acetate to chloride for the conversion of **1** to **1a**, we examined all the mechanisms outlined in Scheme 4. Similar to the reaction between **1** and DCE, the reaction between **1a** and DCE showed the same competitive reaction pathways with the Pt nucleophilic and direct substitution pathways being most viable (see Supporting Information). Importantly, the barriers for these reaction pathways with **1a** are ∼4–6 kcal·mol^–1^ higher in energy than the reaction pathways with **1**, which is consistent with the observation that **1a** is observed as an intermediate from the conversion of **1** to **1b**.

Similar trends are observed for complex **2** with Pt nucleophilic attack and direct substitution pathways being the most viable albeit with increased barriers (16 kcal·mol^–1^ for **TS-B** and 36 kcal·mol^–1^ for **TS-C**). The larger calculated barriers for these transition states are consistent with the experimentally observed difference in rate between complexes **1** and **2**. However, our calculations significantly overestimate this energy difference. Since the calculations indicate that the uncoordinated quinolines in **1** and **2** do not affect the transition state barriers it is likely that the difference in electron donation from the extra quinoline ligand in **1** versus the phenyl ligand in **2** alter the electron density on Pt thereby leading to the observed rate difference.

Then, we examined the difference in energy between Sb containing complex **1** and non-Sb containing complex **3**. Calculated enthalpies suggest an increase in barriers for the acetoxylation of DCE with a slight preference for **TS-D**. **TS-D** is uphill by 4 kcal·mol^–1^ for **3** versus **1**, which is consistent with experimentally observed *k*_obs_ (see Supporting Information Scheme S1). The calculated barriers are higher than expected, which could be due to the calculations overestimating the energy difference or **3** could acetoxylate DCE through a different mechanism than **1**. Altogether, these data in conjunction with experimental data provide evidence for faster rates of DCE acetoxylation for Sb containing Pt bis-acetate complexes versus a non-Sb containing Pt bis-acetate complex. To provide qualitative insight into the reactivity difference between **1** and **3**, we calculated the natural bond orbital (NBO) charges of complexes **1** and **3** as well as the transition states shown in Figure 3. While these complexes showed a similar change in charge at the Pt center going from the ground state to transition states, the starting charges were markedly different. The charge of the Pt center in **1** is 0.3 and 0.7 for **3**. A similar difference was also found with Mulliken charges. This suggests that the Sb containing complex provides a more electron rich Pt center that enables faster reaction through an oxidative addition/reductive elimination process.

### Experimental Analysis of Reactivity of (SbQ_3_)Pt(OAc)_2_ (**1**) and CD_2_Cl_2_ and CDCl_3_

**Figure 4:**
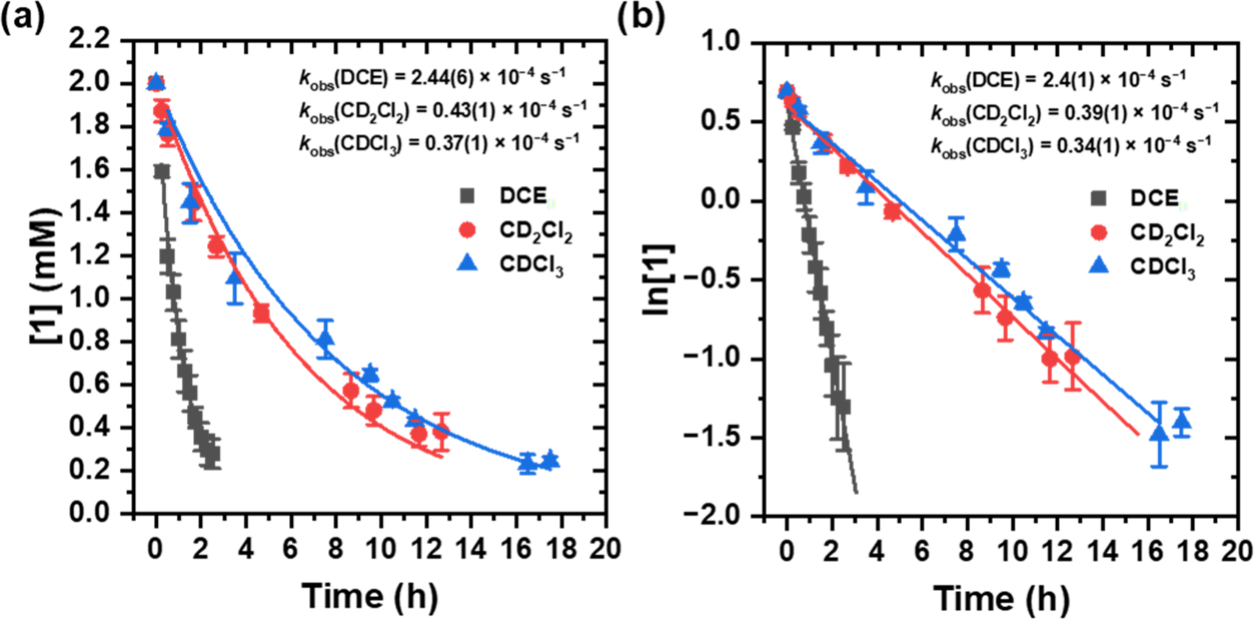
Kinetic studies of the acetoxylation of DCE, CD_2_Cl_2_, and CDCl_3_ using complex **1** at 80 °C. (a) Plot of concentrations of complexes **1** and **1a** versus time. (b) Plot of ln[**1**] versus time.

Given the reactivity with DCE, a primary alkyl chloride, we sought to investigate the reactivity of **1** with dichloromethane*-d*_2_ (CD_2_Cl_2_) and chloroform*-d* (CDCl_3_) in order to explore various substituent effects on the acetoxylation reaction (Figure 4). The reactions were monitored in the same manner as with DCE. The acetoxylation of CD_2_Cl_2_ was monitored over 12.5 h. The yield of the reaction between **1** and CD_2_Cl_2_ was determined by ^1^H NMR spectroscopy to be 74(3)% of **1a** with 19(4)% of **1** remaining with a *k*_obs_ of 0.43(1) × 10^–4^ s^–1^ (calculated via first-order exponential fitting). The acetoxylation of CDCl_3_ was monitored for 17.5 h. The yield of the reaction between **1** and CDCl_3_ was determined by ^1^H NMR spectroscopy to be 67(1)% for **1a** with 12(1)% of **1** remaining with a *k*_obs_ of 0.37(1) × 10^–4^ s^–1^ (calculated via first-order exponential fitting). The observed rate constants follow the trend DCE ≫ CD_2_Cl_2_ ∼ CDCl_3_. The decreased rate with both CD_2_Cl_2_ and CDCl_3_ is likely due to increased stability toward nucleophilic attack through stabilization of the antibonding orbital of the C–Cl bond by neighboring chloride substituents.^54^ The lower reactivity of CDCl_3_ versus CD_2_Cl_2_ could be due to increased steric bulk at carbon.

## Summary and Conclusions

The acetate complexes **1** and **2** react with 1,2-dichloroethane (DCE) to give acetoxylated products and Pt dichloride complexes **1b** and **2b**, respectively. The relative rates of these reactions were probed, and a statistically significant ligand effect was quantified indicating complex **1** reacts with DCE approximately 5 times faster than **2** under pseudo-first order conditions at 80 °C. The non-Sb containing Pt acetate complex **3** was synthesized and studied for the acetoxylation of DCE to provide a comparison with **1** and **2**. The overall mechanism of the reaction with DCE were evaluated via DFT calculations and provided evidence that the ligand effect is likely due to electronic differences between the substituents on the Sb ligands affecting the electron density on Pt. The Pt nucleophilic attack mechanism is supported by the calculations (Scheme 4, **TS-B**); however, there is only a 4 kcal·mol^–1^ preference for this mechanism versus the direct substitution by an acetate ligand (Scheme 4, **TS-D**). The monochloride intermediate **1a** was isolated independently, and the solid-state structure combined with NMR studies provided evidence for a significant through space interaction between coordinated Cl^–^ and a coordinated quinoline proton. Bipyridine based Pt bis-acetate complex **3** was synthesized and studied for the acetoxylation to provide a comparison with a non-Sb containing Pt acetate complex. Both experimental and DFT studies provide evidence for a slower reaction with **3** than Sb containing complex **1** and a similar rate as **2**. The rate of acetoxylation of CD_2_Cl_2_ and CDCl_3_ using complex **1** was studied demonstrating the ability to substitute more sterically bulky alkyl chlorides (albeit at a slower rate). This work demonstrates that while a variety of mechanisms are accessible to multifunctional ligands, the electronic difference between Sb aryl substituents (quinolinyl versus phenyl substituents) is the primary cause of differences in the nucleophilic reactivity of **1** and **2**.

## Experimental Section

### General Information

Complexes **1** and **2** were synthesized as previously reported.^6^ Reactions performed on the NMR scale used Wilmad medium wall precision low pressure/vacuum (LPV) NMR tubes or J. Young tubes purchased from the Yale University Scientific Glassblowing Laboratory. Tetrahydrofuran (THF) and diethyl ether (Et_2_O) were dried using a sodium-benzophenone/ketyl still under a dinitrogen atmosphere and stored over activated 4 Å molecular sieves inside a dinitrogen filled glovebox. Pentane and methylene chloride were dried using a solvent purification system with activated alumina and stored over activated 3 Å molecular sieves inside a dinitrogen filled glovebox. Glovebox purity was maintained through periodic dinitrogen purges and was monitored by water and dioxygen analyzers (O_2_ < 20 ppm and H_2_O < 20 ppm). Chloroform-*d*_1_ and methylene chloride-*d*_2_ were stored over activated 4 Å molecular sieves inside a dinitrogen filled glovebox. All other chemicals were purchased from commercial sources and used as received.

NMR spectra were recorded on a Varian VNMRS (600 MHz), or Bruker Avance III (600 or 800 MHz), or Bruker NEO NanoBay 400 MHz spectrometer. All reported chemical shifts were referenced to residual ^1^H resonances (^1^H NMR) or ^13^C{^1^H} resonances (^13^C{^1^H} NMR). ^1^H NMR: chloroform-*d* 7.26 ppm; methylene chloride*-d*_2_ 5.32 ppm. ^13^C{^1^H} NMR: chloroform-*d* 77.16 ppm; methylene chloride*-d*_2_ 53.84 ppm.^55^

### Synthesis and Characterization of Complex **1a**

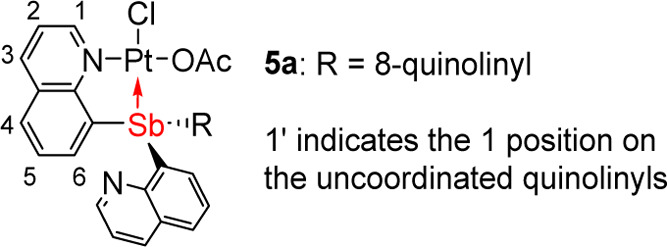



#### (SbQ_3_)PtCl(OAc) (**1a**)

In a pressure tube, complex **1** (84 mg, 0.10 mmol) was dissolved in 5 mL DCE and stirred at 100 °C for 1 h. After cooling to room temperature, the reaction solution was transferred to a 100 mL round-bottom flask and evaporated to yield a yellow residue. The residue was then dissolved in minimal DCM and added to diethyl ether to precipitate a solid. The solid was washed further with diethyl ether then dried under vacuum to yield a white/yellow powder (23 mg, 28% yield), which contained ∼10% of the dichloride product (SbQ_3_)PtCl_2_ (**3**) as an impurity. Single crystals suitable for X-ray diffraction were found after allowing diethyl ether to vapor diffuse into solutions of CDCl_3_ containing **1a** at −15 °C. ^1^H NMR (800 MHz, CDCl_3_) δ 11.25 (dd, ^3^*J*_HH_ = 6 Hz, 2 Hz, 1H, 1-position), 8.61 (dd, ^3^*J*_HH_ = 7 Hz, 1 Hz, 1H, 4- or 6-position), 8.52 (dd, ^3^*J*_HH_ = 4 Hz, 2 Hz, 2H, 1′-position), 8.41 (dd, ^3^*J*_HH_ = 7 Hz, 1 Hz, 2H, 4′- or 6′-position), 8.31 (dd, ^3^*J*_HH_ = 8 Hz, 2 Hz, 1H, 3-position), 8.17 (dd, ^3^*J*_HH_= 8 Hz, 2 Hz, 2H, 3′-position), 7.95 (dd, ^3^*J*_HH_ = 8 Hz, 1 Hz, 2H, 4′- or 6′- position), 7.64 (d, 8 Hz, 1H 4- or 6-position), 7.60 (t, ^3^*J*_HH_ = 8 Hz, 8 Hz, 2H, 5′-position) 7.39 (dd, ^3^*J*_HH_ = 8 Hz, 6 Hz, 1H, 2-position) 7.33 (dd, ^3^*J*_HH_ = 8 Hz, 4 Hz, 2H, 2′-position), 7.25 (m, 1H, 5-position), 2.04 (s, 3H, acetate–C*H*_3_). ^13^C{^1^H} NMR (201 MHz, CDCl_3_) δ 179.3, 158.8, 150.9, 149.7, 149.3, 145.4, 139.7, 139.4, 139.0, 137.0, 136.3, 130.3, 129.7, 129.6, 128.5, 127.8, 127.2, 122.0, 121.6, 19.6. (^13^C{^1^H} resonances corresponding to complex **3** impurity were excluded. Full spectrum containing impurity peaks is shown in the Supporting Information). We were unable to obtain satisfactory elemental analysis of this product.
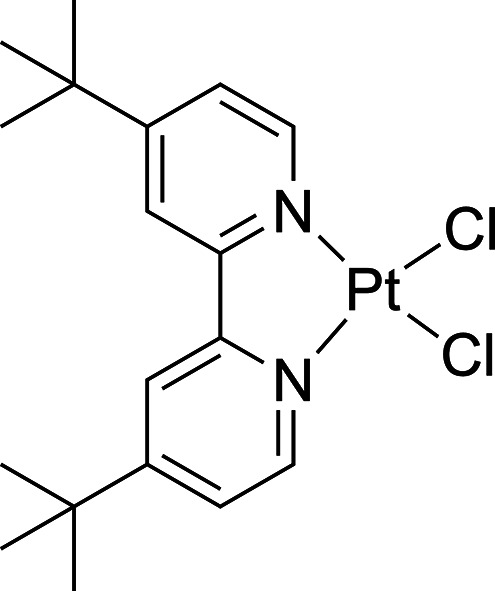


#### (^t^bpy)PtCl_2_

To a pressure tube, 110 mg (0.40 mmol) of PtCl_2_ and 110 mg (0.40 mmol) of ^t^bpy were added. Next, 5 mL of MeCN were added, and the tube was sealed and heated to 85 °C while stirring overnight. The next day the tube was returned to room temperature, the reaction was transferred to a round-bottom flask, and the solvent was reduced in vacuo to leave a yellow residue. The residue was redissolved in DCM and filtered through a Celite-packed frit, and the yellow filtrate was collected. The filtrate was reduced leaving a yellow residue. The remaining residue was redissolved in minimal DCM, and diethyl ether was added to give a yellow precipitate. The yellow powder was washed with diethyl ether and dried under vacuum leaving 170 mg (80% yield). ^1^H NMR (600 MHz, CDCl_3_) δ 9.51 (dd, ^3^*J*_HH_ = 6 Hz, ^4^*J*_HH_ = 2 Hz, 2H), 7.88 (d, ^4^*J*_HH_ = 2 Hz, 2H), 7.50 (dd, ^3^*J*_HH_ = 6, ^4^*J*_HH_ 2 Hz, 2H), 1.46 (s, 18H). The ^1^H NMR spectrum in CDCl_3_ of the obtained powder matched previously reported data.^48^
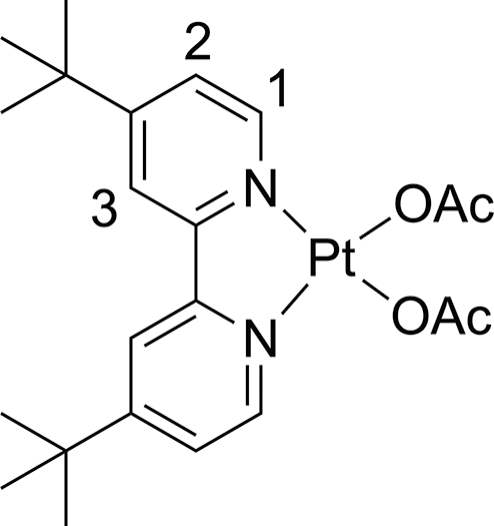


#### (^t^bpy)Pt(OAc)_2_ (**3**)

To a round-bottom flask, 110 mg (0.20 mmol) of (^t^bpy)Pt(Cl)_2_ and 86 mg (0.50 mmol) of AgOAc were added in darkness and wrapped in foil. The solids were dispersed with ∼30 mL DCM and stirred at room temperature for 2 days. The red/brown reaction was filtered through a Celite-packed frit, and the filtrate was reduced in vacuo. The remaining residue was redissolved in minimal DCM, and diethyl ether was added to give a brown precipitate. The brown powder was washed with diethyl ether and dried under vacuum to give 56 mg (48% yield). Single crystals suitable for X-ray diffraction were found after allowing diethyl ether to vapor diffuse into solutions of CDCl_3_ containing **3** at −15 °C. ^1^H NMR (600 MHz, CDCl_3_) δ 8.68 (d, ^3^*J*_HH_ = 6 Hz, 2H, 1-position), 7.77 (d, ^4^*J*_HH_ = 2 Hz, 2H, 3-position), 7.49 (dd, ^3^*J*_HH_ = 6, ^4^*J*_HH_ = 2 Hz, 2H, 2-position), 2.19 (s, 6H, acetate–C*H*_3_), 1.42 (s, 18H, *tert*-butyl–C*H*_3_). ^13^C{^1^H} NMR (201 MHz, CDCl_3_) δ 178.4, 164.3, 156.9, 150.0, 124.2, 118.8, 36.1, 30.3, 23.6. We were unable to obtain satisfactory elemental analysis of this product.

### Kinetics Studies (DCE)

To a J-Young tube with sealed capillaries containing CDCl_3_, 0.4 mL of a stock solution containing 2.0 mM complex **1**, **2**, or **3** and 2.0 mM 1,3,5-trimethoxybenzene in DCE was added (∼6300 equiv of DCE). The tubes were heated at 80 °C in an oil bath and the reaction was monitored in 15 min intervals totaling 150 min for **1**, in 60 min intervals totaling 10 h for **2**, and 11.5 h for **3** using 9 time points at various intervals via ^1^H NMR spectroscopy using a solvent suppression pulse sequence to presaturate the protio-DCE peak. The concentration of **1**, **2**, and **3** were calculated using the integration ratio to the internal standard proton resonance at 6 ppm. The reactions have been performed in triplicate for each condition.

### Kinetic Studies (CD_2_Cl_2_ and CDCl_3_)

To a J-Young tube, 0.4 mL of a stock solution containing 2.0 mM complex **1** and 2.0 mM of 1,3,5-trimethoxybenzene in either CD_2_Cl_2_ or CDCl_3_ were added. The tubes were heated at 80 °C in an oil bath, and the reaction was monitored in 10 intervals totaling 12.5 h for CD_2_Cl_2_ and 17.5 h for CDCl_3_ (∼7800 equiv of CD_2_Cl_2_ and ∼6200 equiv of CDCl_3_). The concentration of **1**, **2**, and **3** were quantified using the integration ratio to the internal standard proton resonance at 6 ppm. The reactions were performed in triplicate for each condition.

### Computational Methods

The M06/def2-SVP method and basis set in Gaussian 16 were used to optimize all structures to stationary points characterized as minima or first-order saddles. IRC calculations were used to verify transition-state structure to intermediate connections. The CPCM continuum model for DCE was used for all geometry optimizations and subsequent energy evaluations. Single point calculations with B2PLYP-D3(BJ)/def2-TZVPD were carried out using the ORCA program. All energies are reported relative to starting Pt complex.

## Supporting Information Available

Additional experimental procedures and details, NMR spectra of complexes, crystal structure data, computational details, absolute energies (PDF)
Cartesian coordinates for the calculated structures (XYZ)

## Supplementary Material

om4c00399_si_001.pdf

om4c00399_si_002.xyz
