# Supplementary material for: Mechanistic Studies of Alkyl Chloride Acetoxylation by Pt–Sb Complexes
Source: Organometallics. 2025 Feb 20;44(5):617–27. doi: 10.1021/acs.organomet.4c00399 (PMC11898169; doi:10.1021/acs.organomet.4c00399)
Supplement: Supplementary file 1 — om4c00399_si_001.pdf [file om4c00399_si_001.pdf]

Supporting Information
for

Mechanistic Studies of Alkyl Chloride Acetoxylation by Pt–Sb Complexes

Christopher K. Webber[†], Jugal Kumawat[§], Fanji Kong[†], Diane A. Dickie[†], Daniel H. Ess[§,*] and T. Brent Gunnoe[†,*]

[†]Department of Chemistry, University of Virginia; Charlottesville, Virginia 22904, United States.

[§]Department of Chemistry and Biochemistry, Brigham Young University; Provo, Utah 84604, United States.

*Corresponding authors. Email: tbg7h@virginia.edu, dhe@chem.byu.edu

Table of Contents

page

1. Additional Details for Kinetic Analysis S3
    1.1. Signal-to-noise issue for NMR reactions at low concentrations.................. S3
    1.2. Experimental details for kinetic studies.. S4
2. NMR Spectra S18
3. X-Ray Crystal Structure Data S21
4. DFT Data S23
5. References S38

1. Additional Details for Kinetic Analysis

1.1. Signal-to-noise issue for NMR reactions at low concentrations

During the measurement of observed rate constant k_{obs} for acetoxylation of DCE using $(SbQ_2Ph)Pt(OAc)_2$ (**2**) at 80 °C, a large deviation was observed for the last 3 to 4 time points (**Figure S1**), which is due to the low signal-to-noise ratio of the proton resonances at low concentrations. Specifically, toward the end of trial #3 of the reaction using complex **2** (at 8, 9, and 10 hours), the peak intensity of complex **2** was so low that it was almost buried in the baseline (**Figure S2**, label Y1 and Y3). Therefore, the integrated peak areas at the 8, 9, and 10 hour time points were close to zero and significantly affected by the baseline noise. Thus, we conclude that the obtained integration values of these peaks are inaccurate, leading to the slight curve at the end of the **ln[2]** vs time plot. For trial #3 the last two time points were not used due to the low concentration.

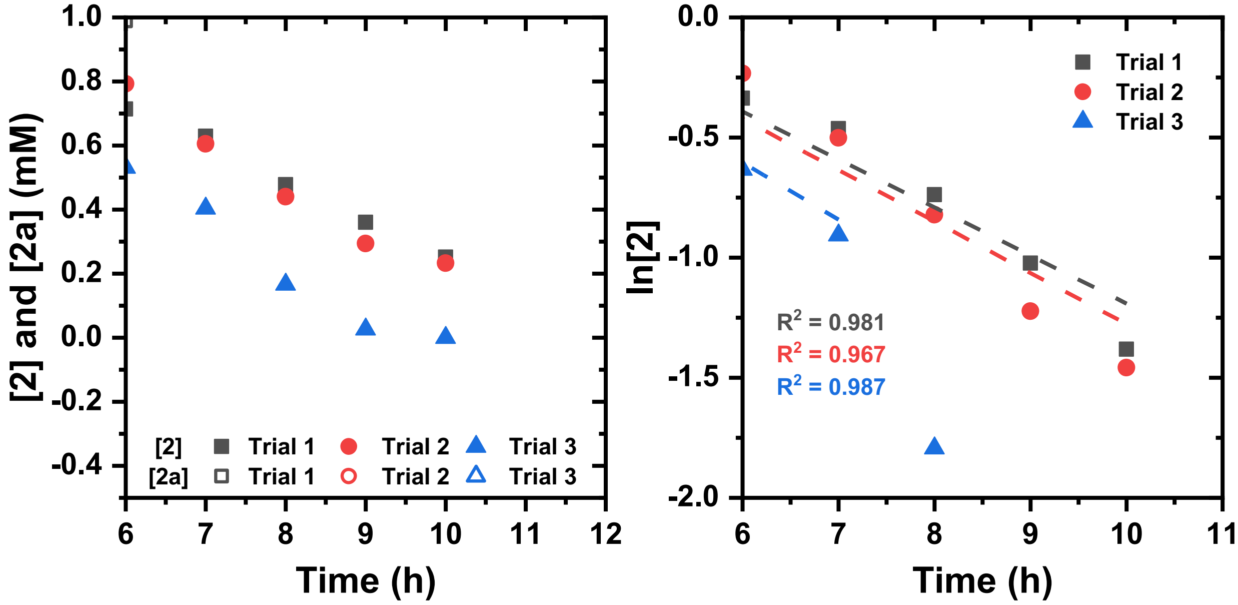

Figure S1. Measurement of observed rate constant k_{obs} for acetoxylation of DCE using complex **2** at 80 °C. *rate* = k_1[**2**][DCE]^X = k_{obs}[**2**], where k_{obs} = k_1[DCE]^X (superscript X denotes that the order in [DCE] is not known).

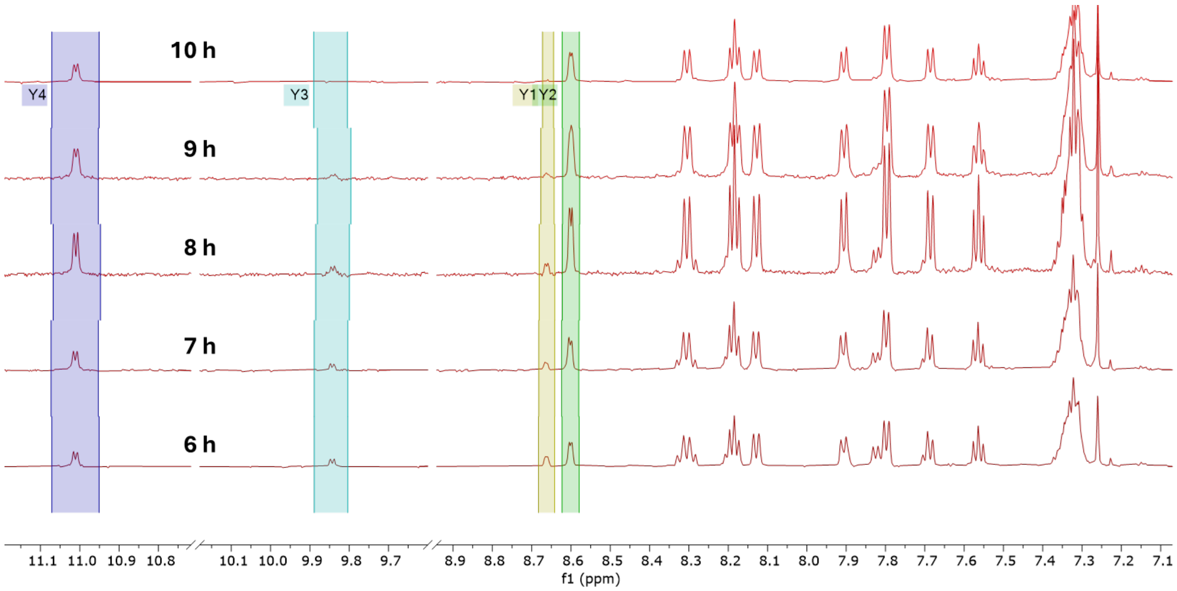

Figure S2. ^{1}H NMR spectra of acetoxylation of DCE using complex **2** at 80 °C

1.2. Experimental details for kinetic studies

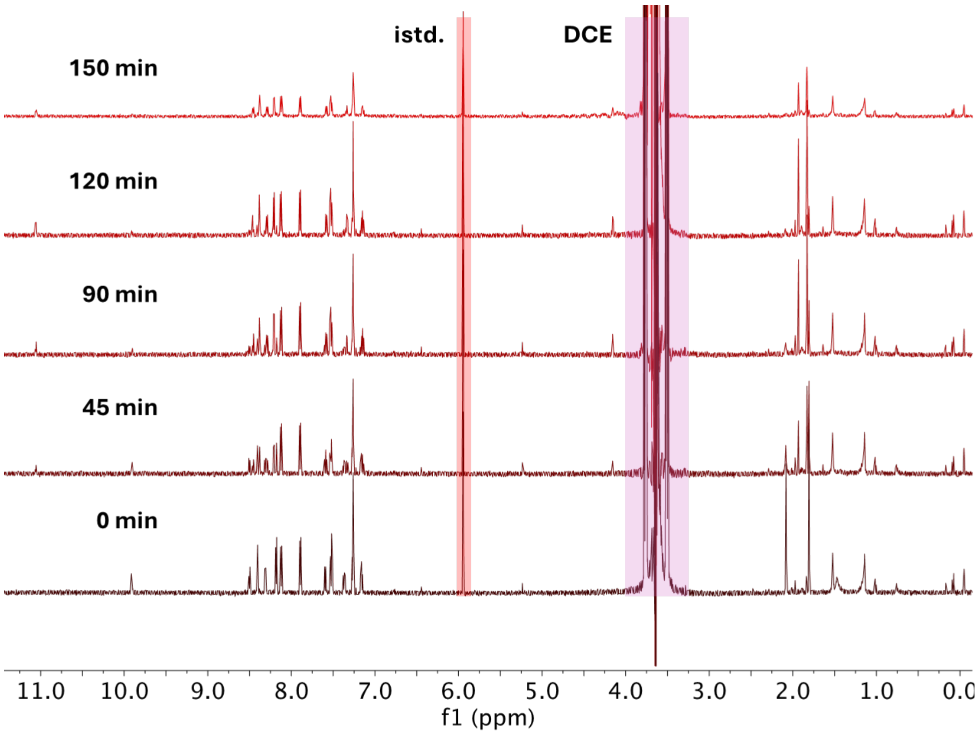

Figure S3. Representative ^{1}H NMR spectra of a kinetic experiment of $(SbQ_3)Pt(OAc)_2$ (**1**) reacting in DCE at 80 ºC to form $(SbQ_3)PtCl(OAc)$ (**1a**) and 2-chloroethylacetate. *Note*: trace water, pentanes and silica grease were present in spectra.

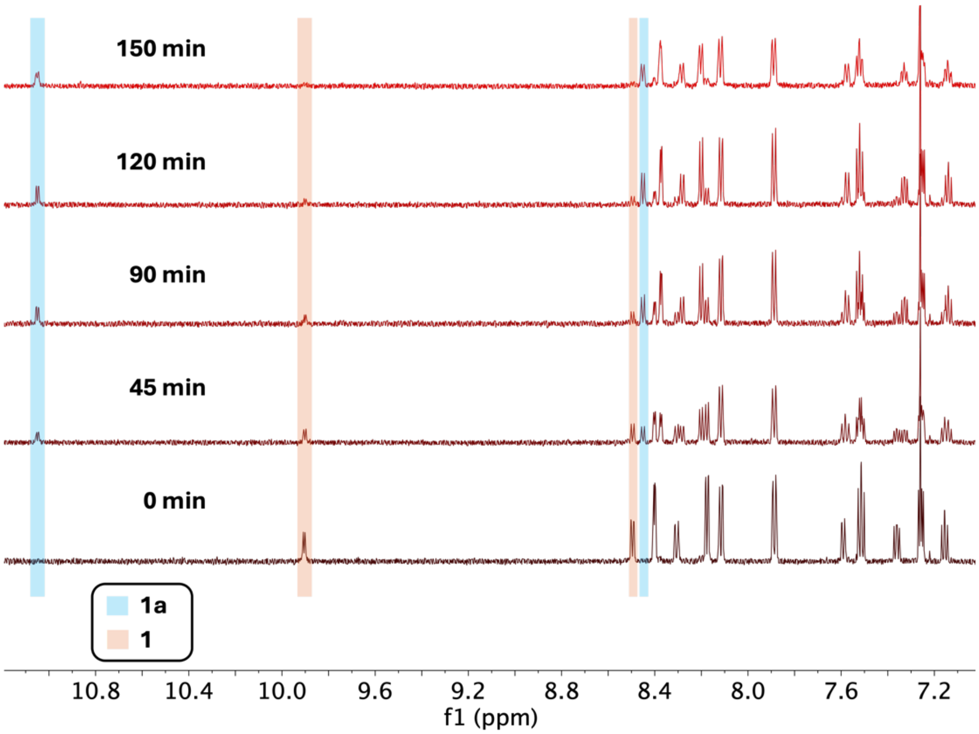

Figure S4. Representative ^{1}H NMR spectra (aromatic region) of a kinetic experiment of $(SbQ_3)Pt(OAc)_2$ (**1**) reacting in DCE at 80 ºC to form $(SbQ_3)PtCl(OAc)$ (**1a**) and 2-chloroethylacetate.

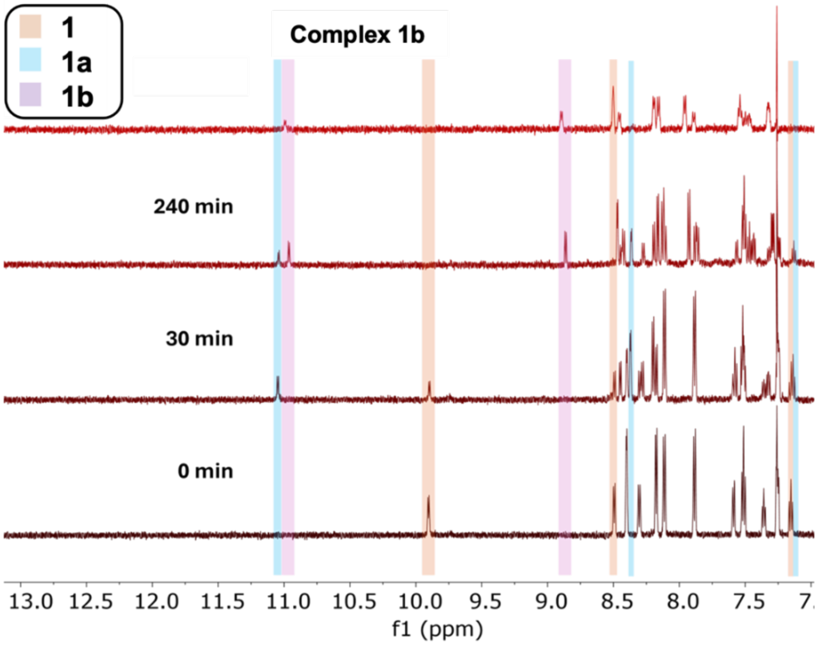

Figure S5. Representative ^{1}H NMR spectra (aromatic region) of an experiment of $(SbQ_3)Pt(OAc)_2$ (**1**) reacting in DCE at 100 ºC with extended time to form $(SbQ_3)PtCl(OAc)$ (**1a**), $(SbQ_3)PtCl_2$ (**1b**) and 2-chloroethylacetate.

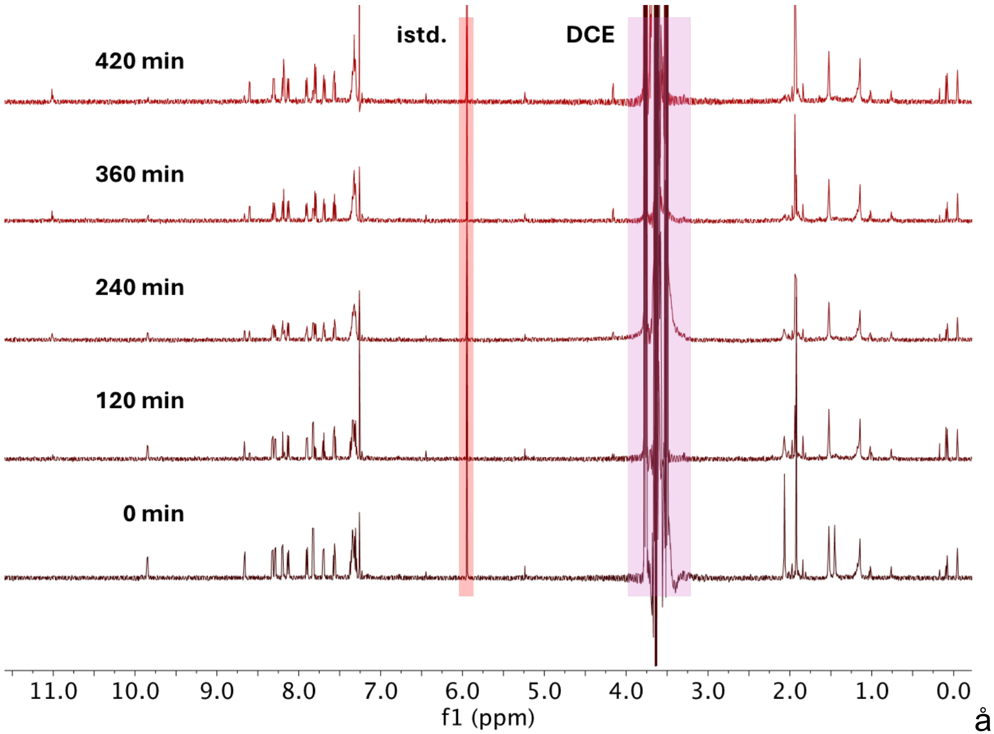

å

Figure S6. Representative ^{1}H NMR spectra of a kinetic experiment of $(SbQ_2Ph)Pt(OAc)_2$ (**2**) reacting in DCE at 80 ºC to form $(SbQ_2Ph)PtCl(OAc)$ (**2a**) and 2-chloroethylacetate. *Note*: trace water, pentanes and silica grease were present in spectra.

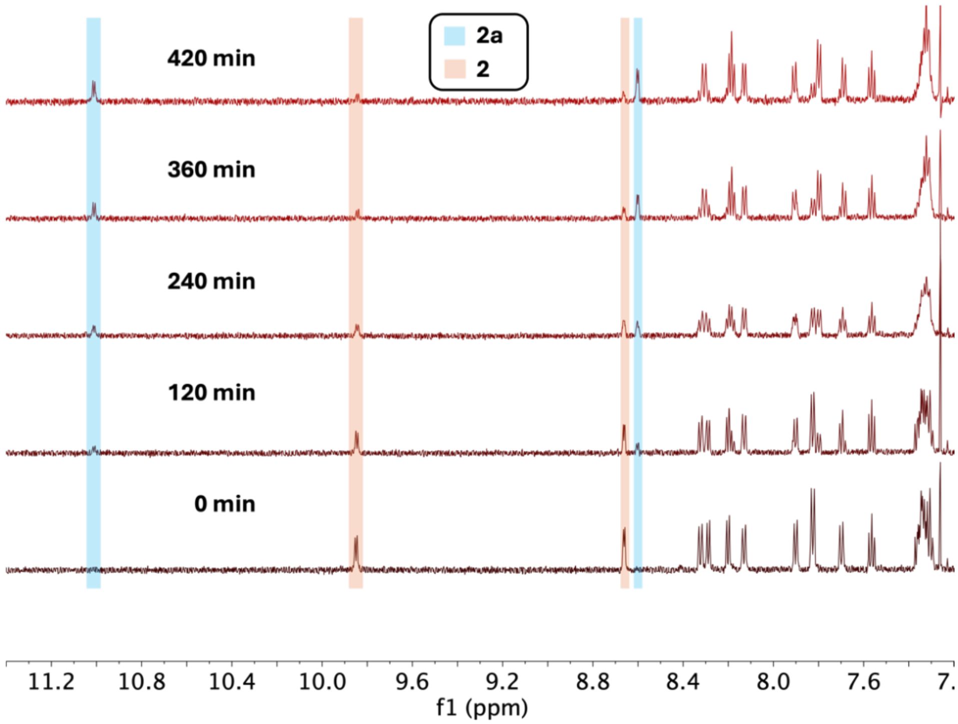

Figure S7. Representative ^{1}H NMR spectra (aromatic region) of a kinetic experiment of $(SbQ_2Ph)Pt(OAc)_2$ (**2**) reacting in DCE at 80 ºC to form $(SbQ_2Ph)PtCl(OAc)$ (**2a**) and 2-chloroethylacetate.

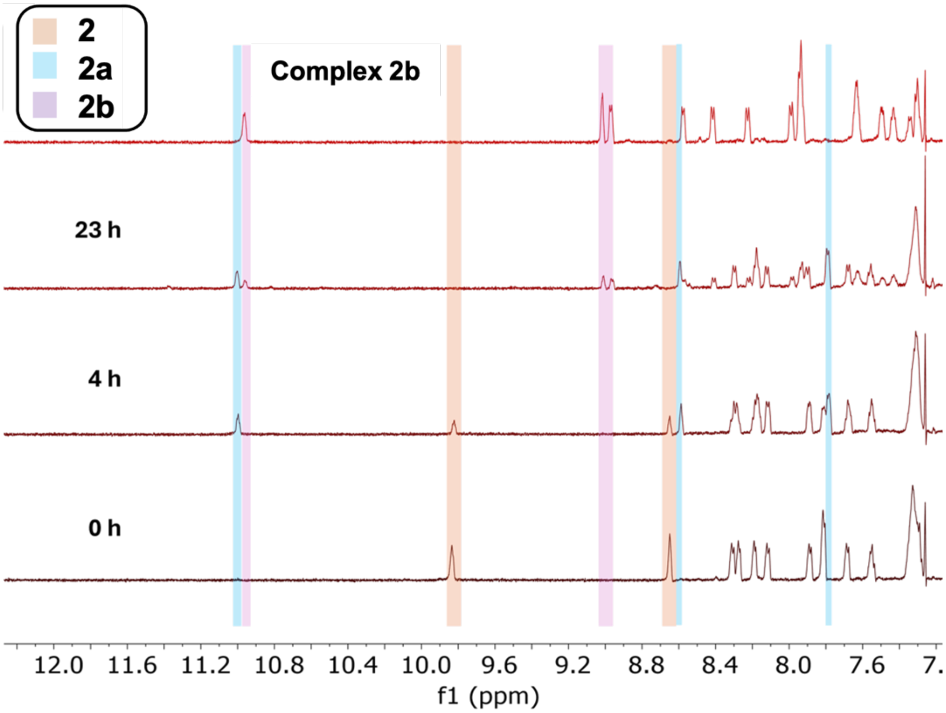

Figure S8. Representative ^{1}H NMR spectra (aromatic region) of (SbQ_2Ph)Pt(OAc)$_2$ (**2**) reacting in DCE at 100 ºC with extended time to form (SbQ_2Ph)PtCl(OAc) (**2a**), (SbQ_2Ph)$PtCl_2$ (**2b**) and 2-chloroethylacetate.

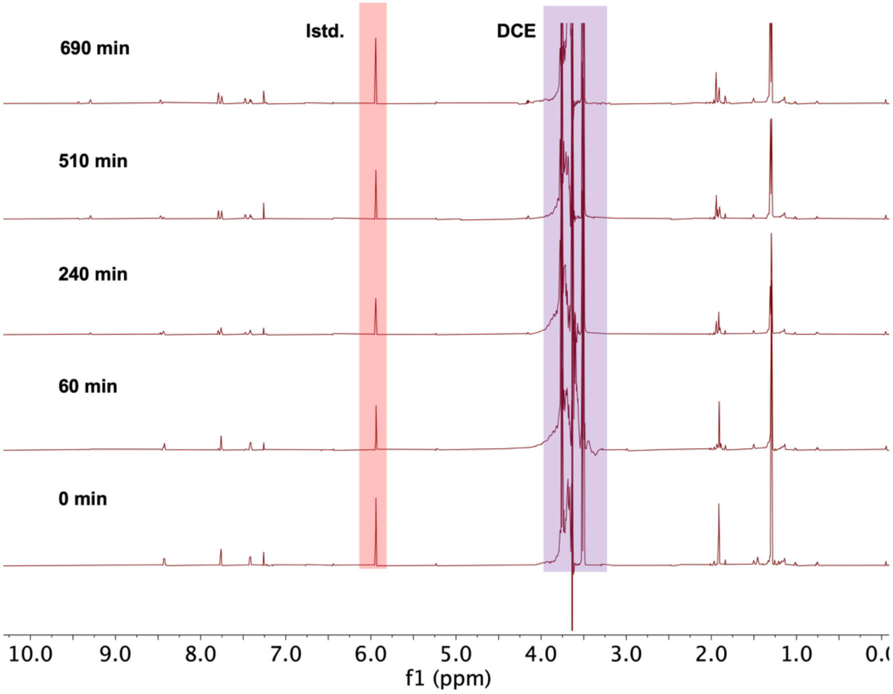

Figure S9. Representative ^{1}H NMR spectra of a kinetic experiment of (^{t}bpy)Pt(OAc)$_2$ (**3**) reacting in DCE at 80 ºC to form (^{t}bpy)PtCl(OAc) (**3a**) and 2-chloroethylacetate. *Note*: trace pentanes and silica grease were present in spectra.

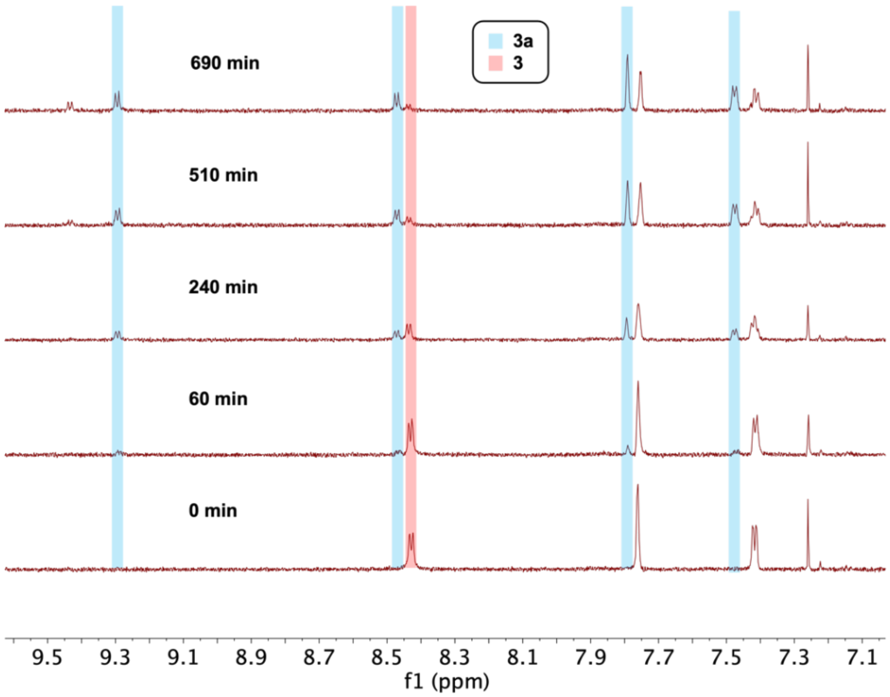

Figure S10. Representative ^{1}H NMR spectra (aromatic region) of a kinetic experiment of (^{t}bpy)Pt(OAc)$_2$ (**3**) reacting in DCE at 80 ºC to form (^{t}bpy)PtCl(OAc) (**3a**) and 2-chloroethylacetate. *Note*: a small amount of an unknown side-product is observed at 9.43 ppm.

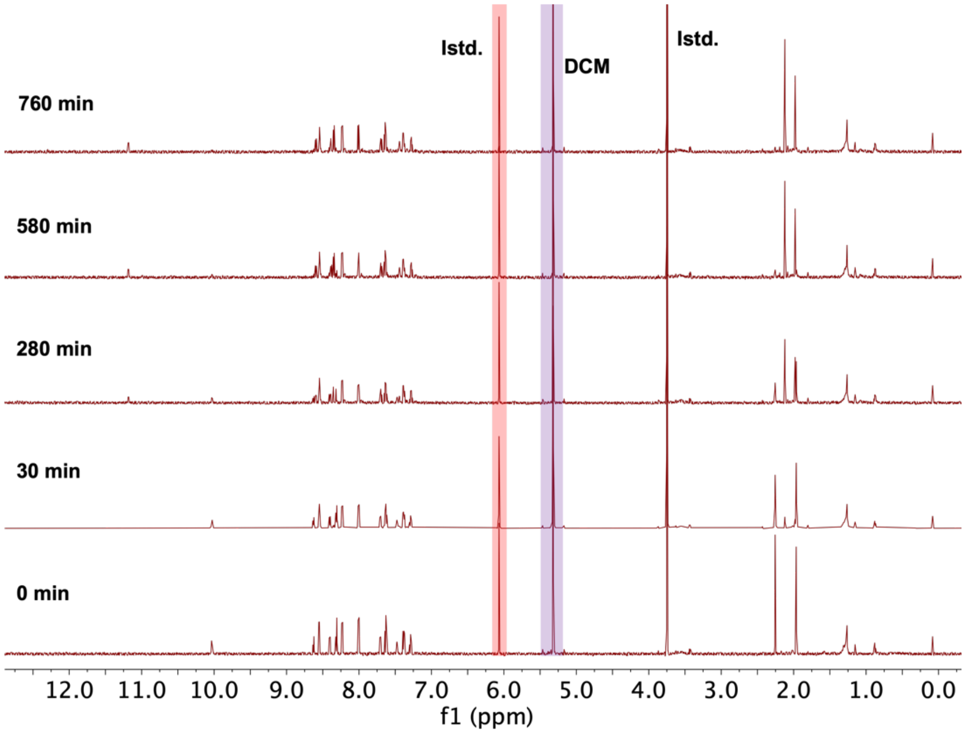

Figure S11. Representative 1H NMR spectra of a kinetic experiment of (SbQ_3)Pt(OAc)$_2$ (**1**) reacting in CD_2Cl_2 at 80 ºC to form (SbQ_3)PtCl(OAc) (**1a**). Note: trace pentanes and silica grease were present in spectra.

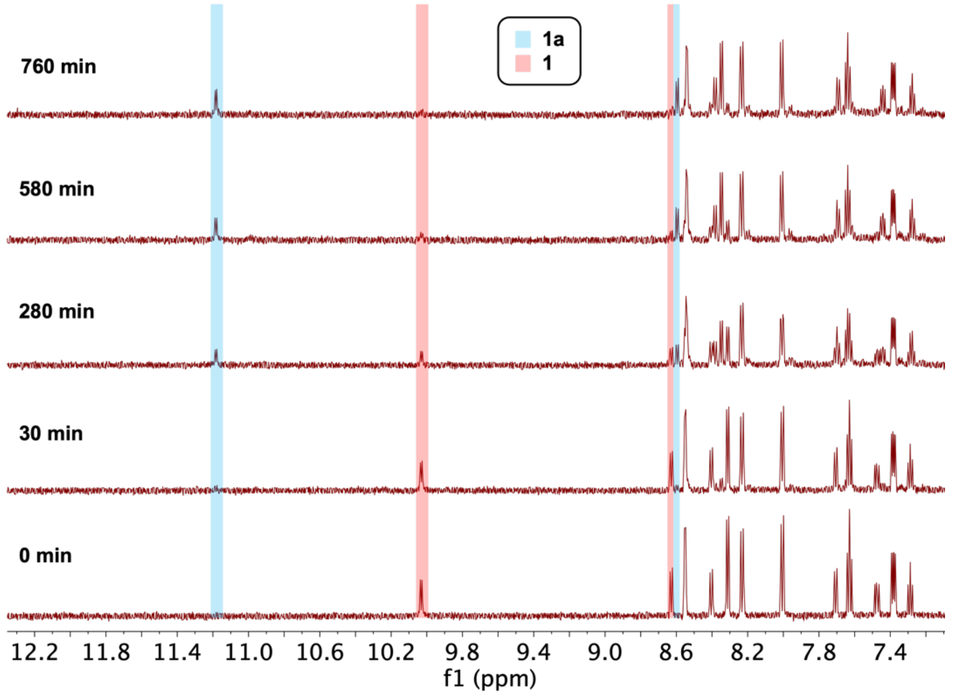

Figure S12. Representative ^{1}H NMR spectra (aromatic region) of a kinetic experiment of $(SbQ_3)Pt(OAc)_2$ (**1**) reacting in CD_2Cl_2 at 80 ºC to form $(SbQ_3)PtCl(OAc)$ (**1a**).

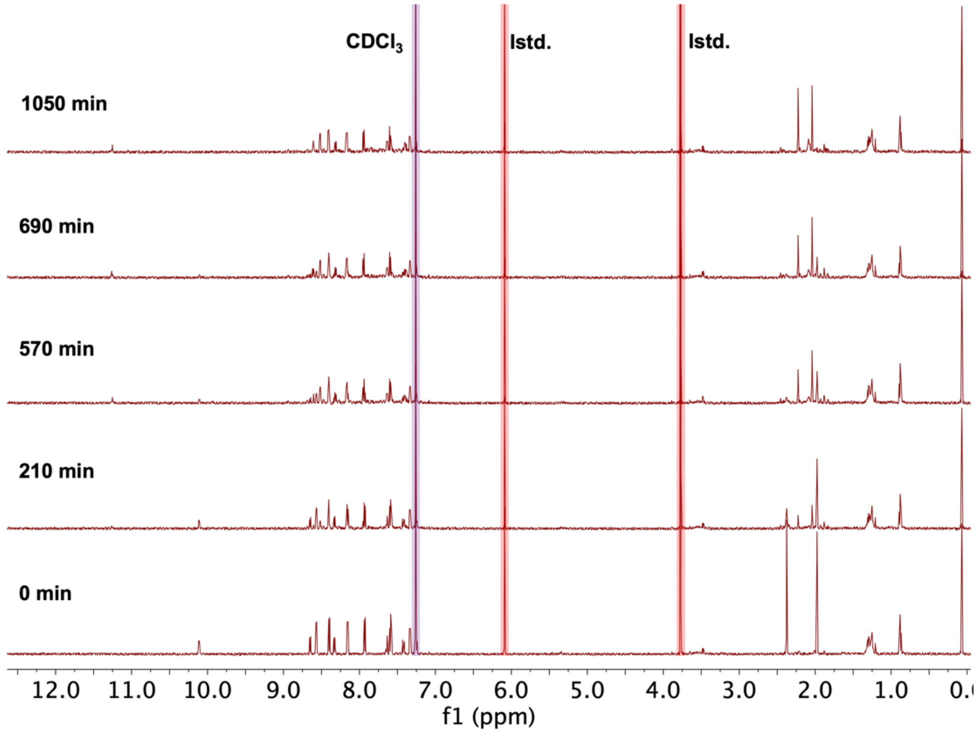

Figure S13. Representative ^{1}H NMR spectra of a kinetic experiment of $(SbQ_3)Pt(OAc)_2$ (**1**) reacting in $CDCl_3$ at 80 ºC to form $(SbQ_3)PtCl(OAc)$ (**1a**). *Note*: trace pentanes and silica grease were present in spectra.

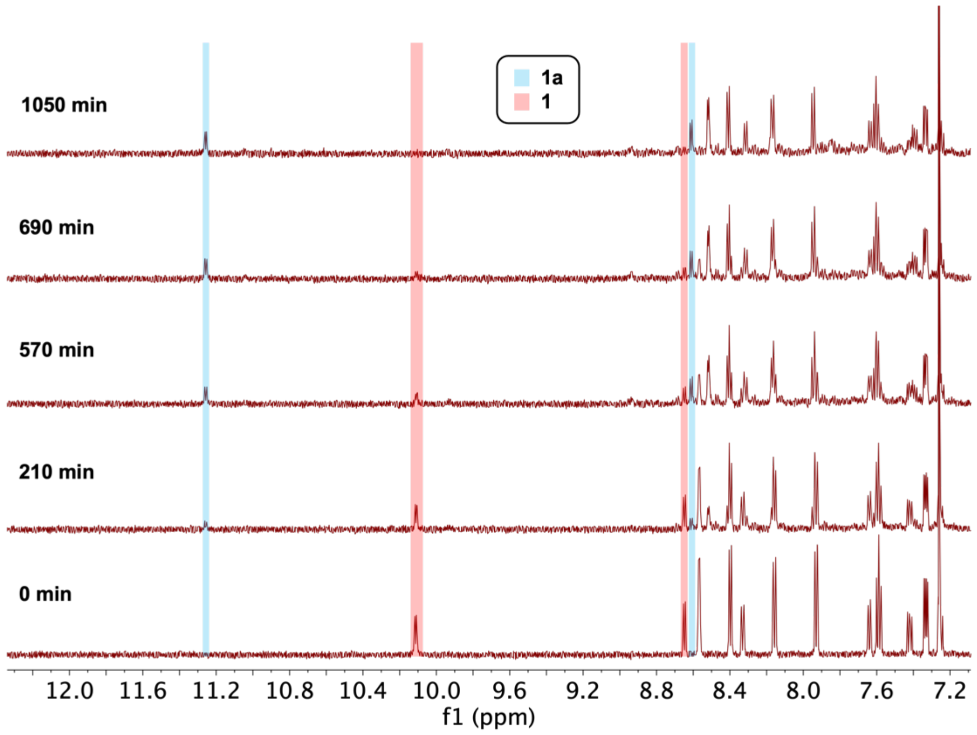

Figure S14. Representative ¹H NMR spectra (aromatic region) of a kinetic experiment of $(SbQ_3)Pt(OAc)_2$ (**1**) reacting in $CDCl_3$ at 80 ºC to form $(SbQ_3)PtCl(OAc)$ (**1a**).

Note: *trace solvent impurities were present in kinetic spectra that we propose do not interfere with the transformation being studied.*

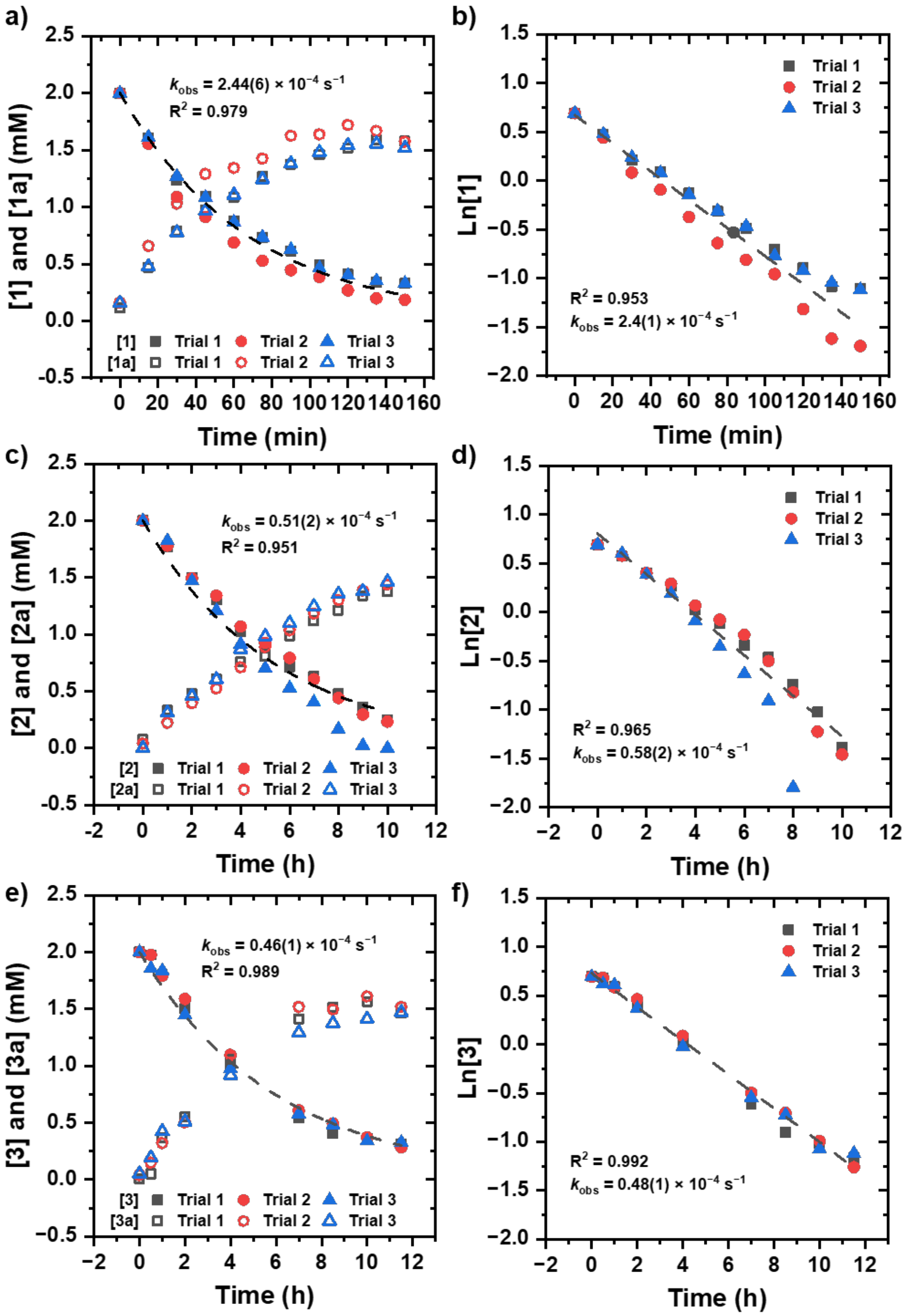

Figure S15. Kinetic studies of the acetoxylation of DCE using complex **1**, **2,** or **3** at 80 °C. The observed rate constant was calculated via a first-order exponential fit and through linearization. All data points from three independent trials have been concatenated as a whole dataset for curve or linear fit. a) Plot of concentrations of complexes **1** and **1a** versus time for the reaction of **1** with DCE. b) Plot of ln[**1**] versus time for the reaction of **1** with DCE. c) Plot of concentrations of complexes **2** and **2a** versus time for the reaction of **1** with DCE. d) Plot of ln[**2**] versus time for the reaction of **2** with DCE. e) Plot of

concentrations of complexes **3** and **3a** versus time for the reaction of **3** with DCE. f) Plot of ln[**3**] versus time for the reaction of **3** with DCE.

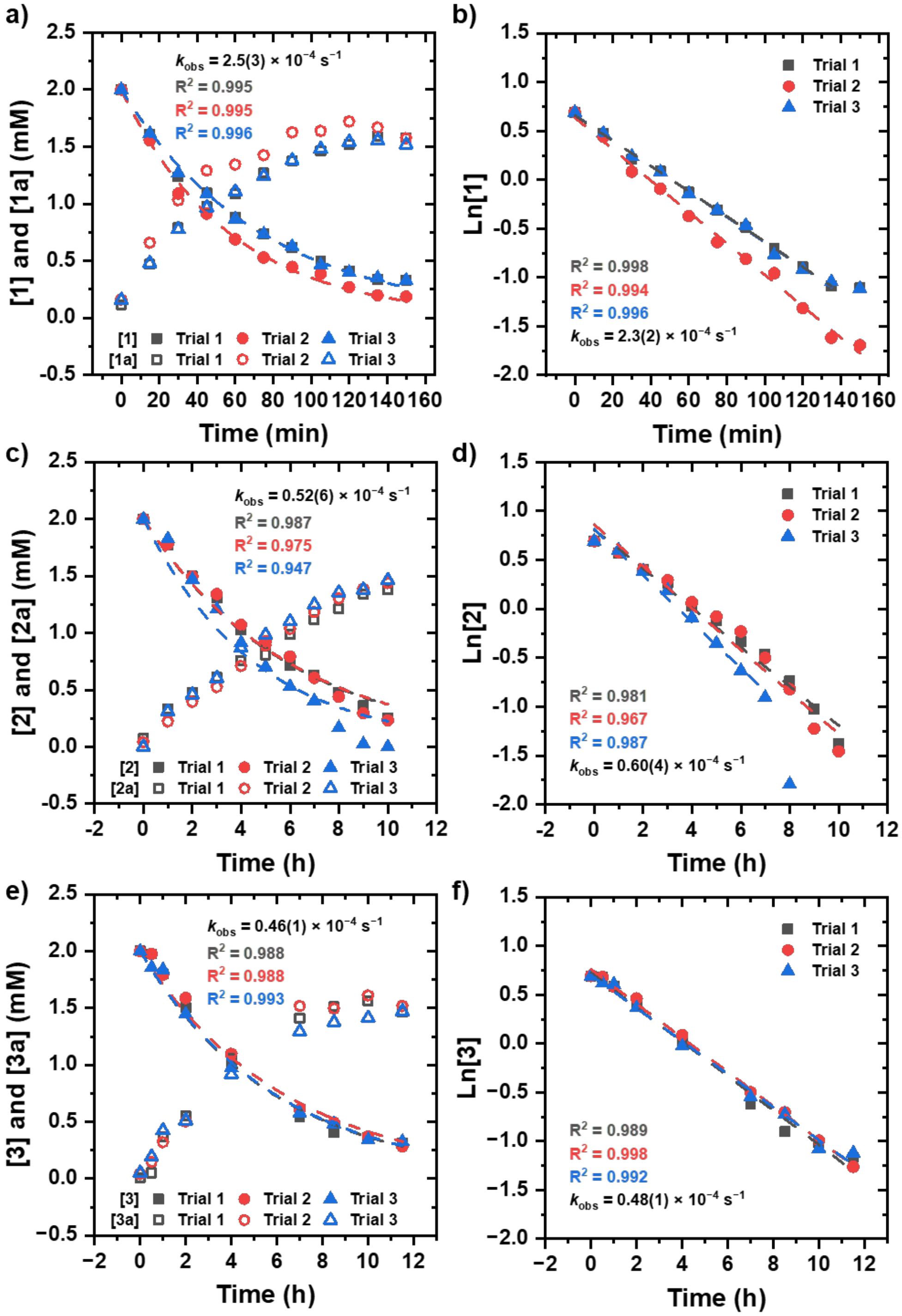

Figure S16. Kinetic studies of the acetoxylation of DCE using complex **1**, **2,** or **3** at 80 °C. The observed rate constant was calculated via a first-order exponential fit and through linearization. The data points from each independent trial have been fitted separately. a) Plot of concentrations of complexes **1** and **1a** versus time for the reaction of **1** with DCE. b) Plot of ln[**1**] versus time for the reaction of **1** with DCE. c) Plot of concentrations of complexes **2** and **2a** versus time for the reaction of **1** with DCE. d) Plot of ln[**2**] versus time for the reaction of **2** with DCE. e) Plot of concentrations of complexes **3** and **3a**

versus time for the reaction of **3** with DCE. f) Plot of ln[**3**] versus time for the reaction of **3** with DCE.

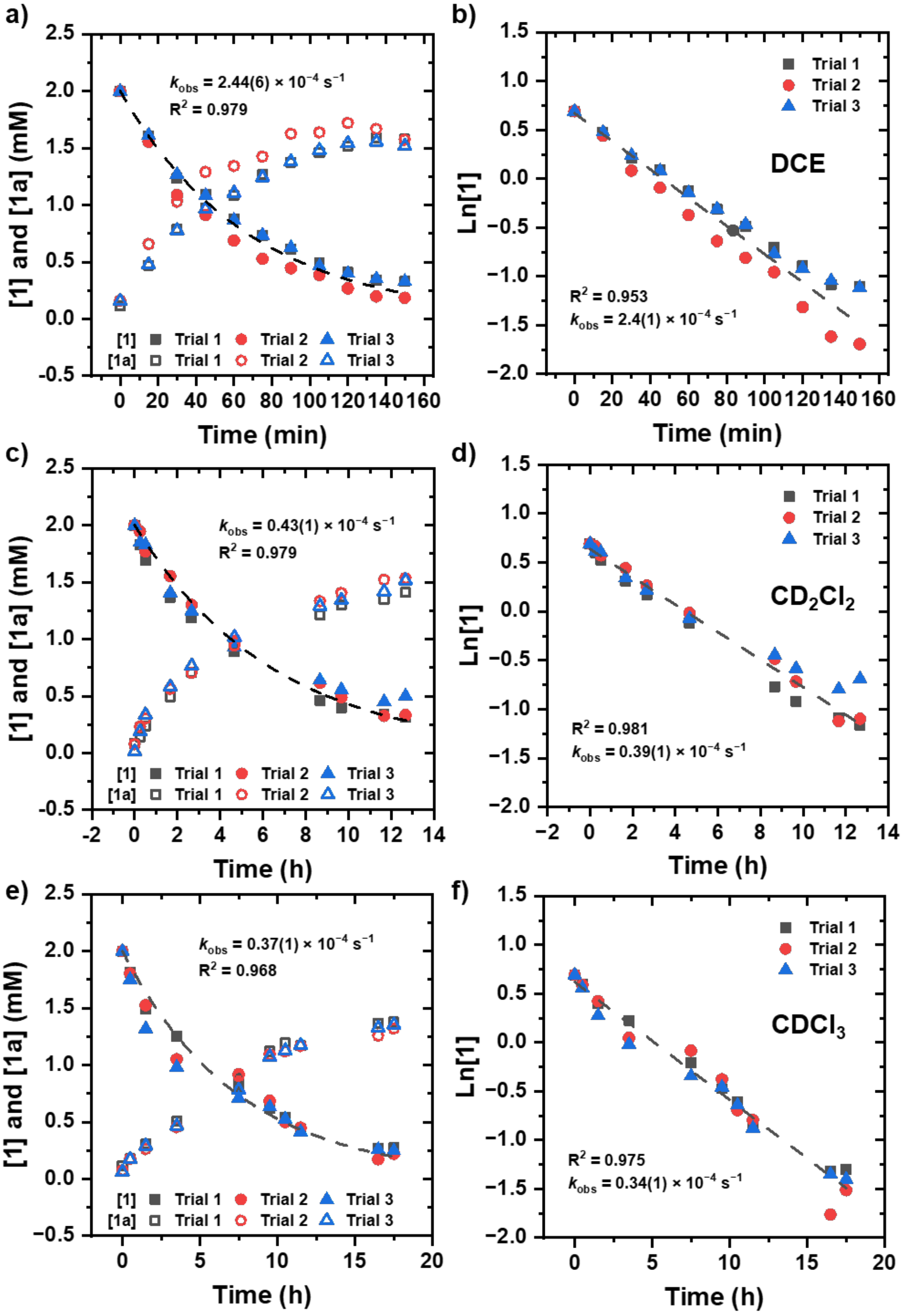

Figure S17. Kinetic studies of the acetoxylation of DCE, CD_2Cl_2 and $CDCl_3$ using complex **1** at 80 °C. The observed rate constant was calculated via a first-order exponential fit and through linearization. All data points from three independent trials have been concatenated as a whole dataset for curve or linear fit. a) Plot of concentrations of complexes **1** and **1a** versus time for the reaction of **1** with DCE. b) Plot of ln[**1**] versus

time for the reaction of **1** with DCE. c) Plot of concentrations of complexes **1** and **1a** versus time for the reaction of **1** with CD_2Cl_2. d) Plot of ln[**1**] versus time for the reaction of **1** with CD_2Cl_2. e) Plot of concentrations of complexes **1** and **1a** versus time for the reaction of **1** with $CDCl_3$. f) Plot of ln[**1**] versus time for the reaction of **1** with $CDCl_3$.

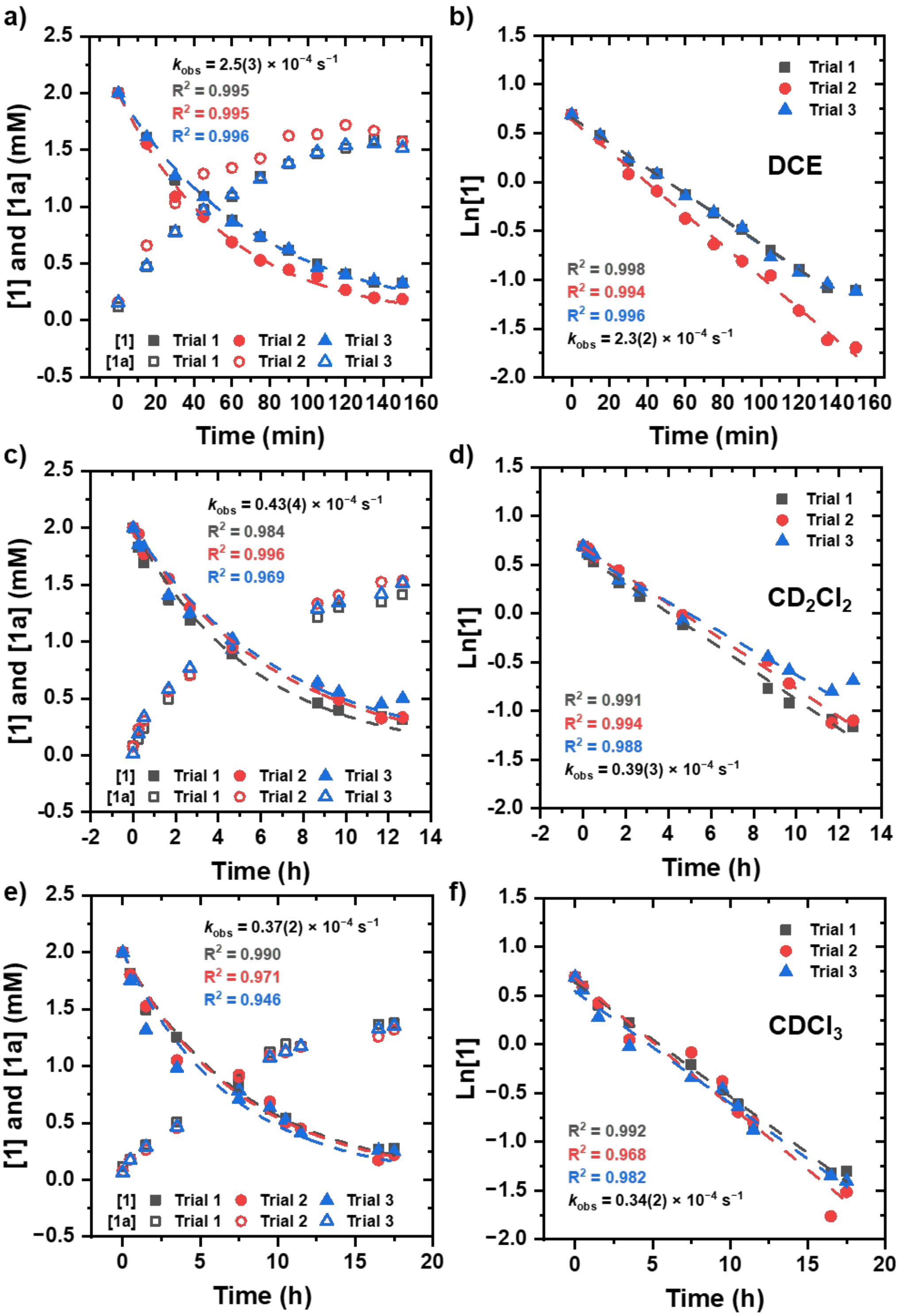

Figure S18. Kinetic studies of the acetoxylation of DCE, CD_2Cl_2 and $CDCl_3$ using complex **1** at 80 °C. The observed rate constant was calculated via a first-order exponential fit and through linearization. The data points from each independent trial have been fitted separately. a) Plot of concentrations of complexes **1** and **1a** versus time for the reaction of **1** with DCE. b) Plot of ln[**1**] versus time for the reaction of **1** with DCE. c) Plot of

concentrations of complexes **1** and **1a** versus time for the reaction of **1** with CD_2Cl_2. d) Plot of ln[**1**] versus time for the reaction of **1** with CD_2Cl_2. e) Plot of concentrations of complexes **1** and **1a** versus time for the reaction of **1** with $CDCl_3$. f) Plot of ln[**1**] versus time for the reaction of **1** with $CDCl_3$.

2. NMR Spectra

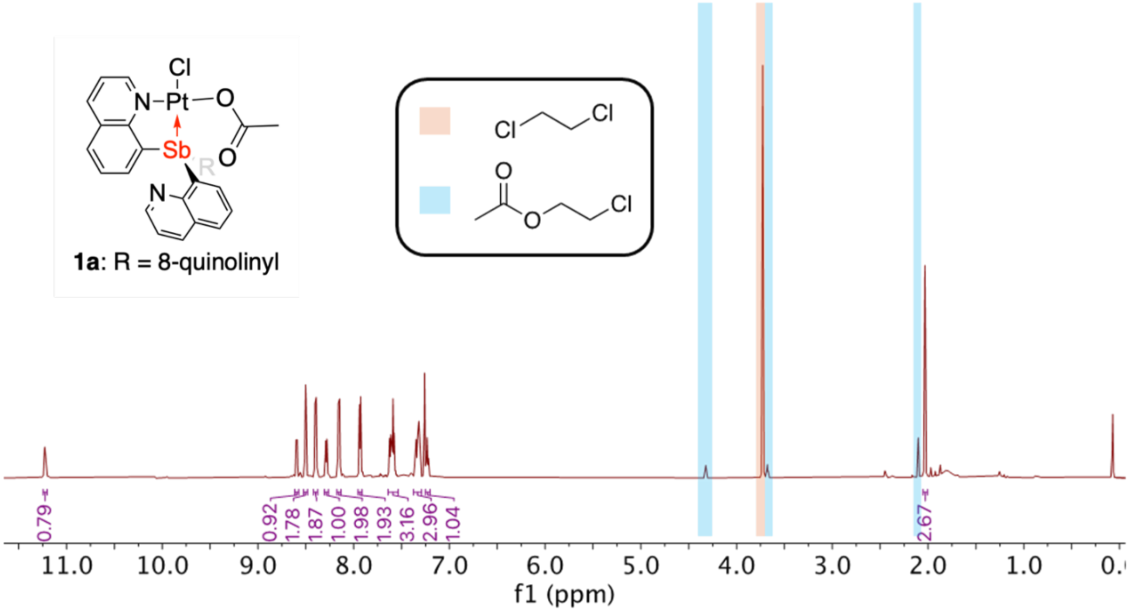

Figure S19. Representative ^{1}H NMR spectrum of $(SbQ_3)PtCl(OAc)$ (**1a**) and 2-chloroethylacetate (600 MHz, $CDCl_3$) from the reaction of $(SbQ_3)Pt(OAc)_2$ (**1**) in DCE .

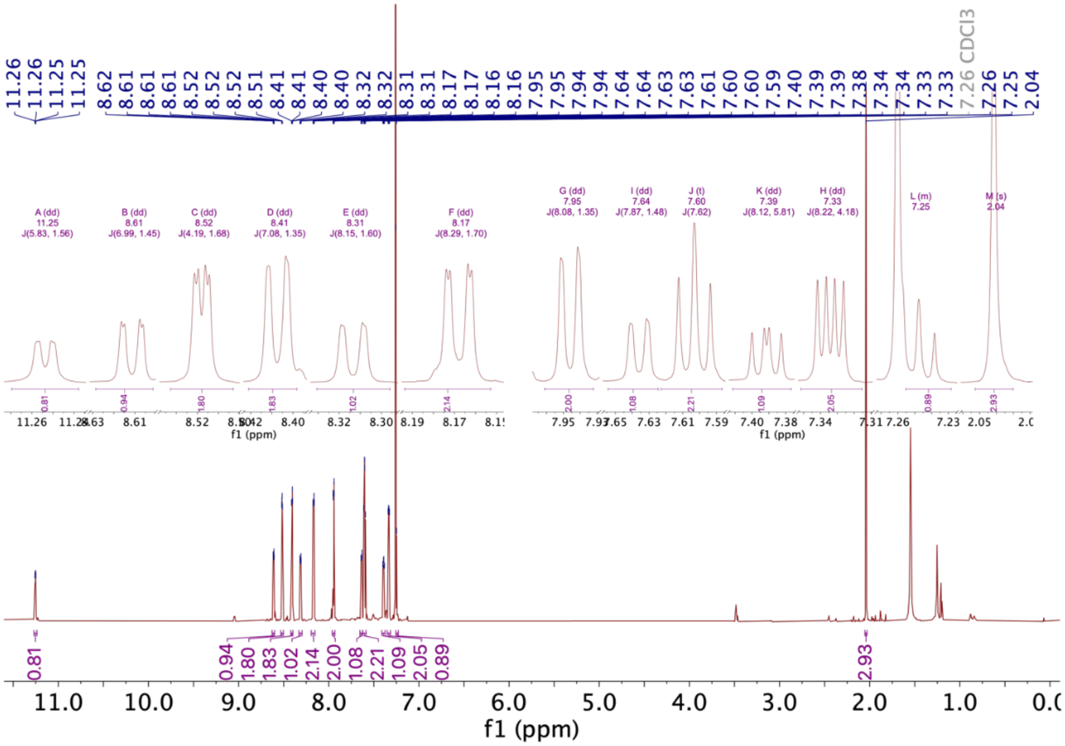

Figure S20. ^{1}H NMR spectrum of $(SbQ_3)PtCl(OAc)$ (**1a**). (800 MHz, $CDCl_3$). *Note*: product contained $(SbQ_3)PtCl_2$ (**3**) as an impurity. Water, diethyl ether, and H-grease impurities were present in the spectrum.

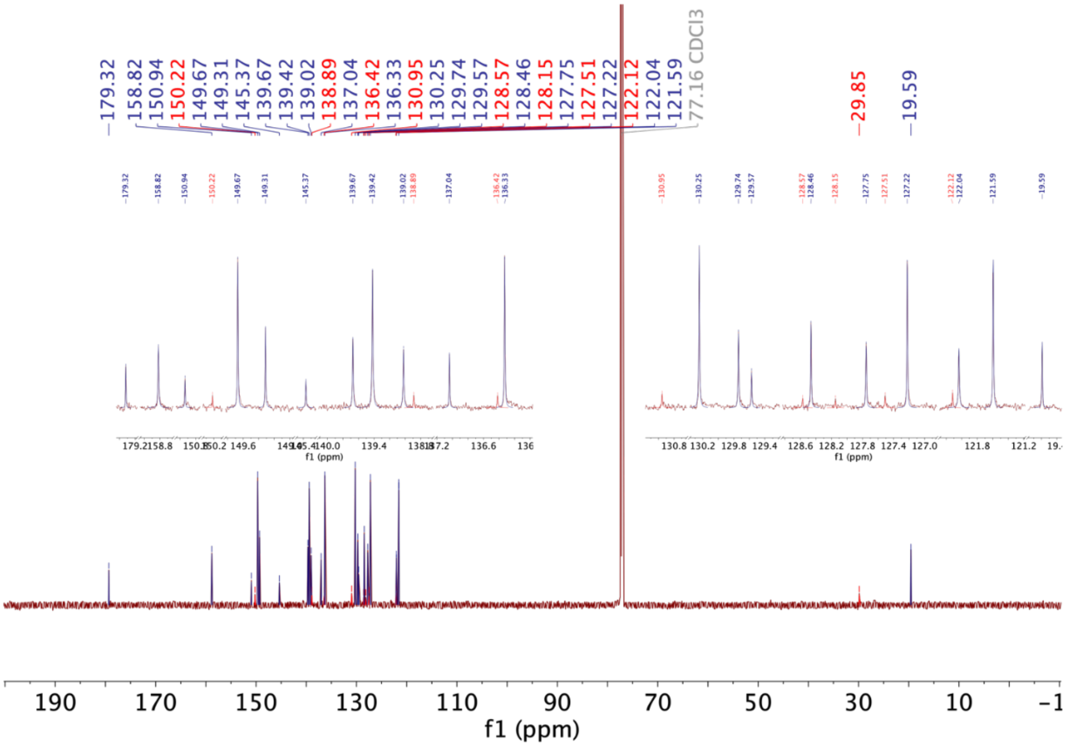

Figure S21. ^{13}C{^{1}H} NMR spectrum of $(SbQ_3)PtCl(OAc)$ (**1a**). (201 MHz, $CDCl_3$). *Note*: product contained $(SbQ_3)PtCl_2$ (**3**) as an impurity leading to more ^{13}C{^{1}H} resonances

than expected. $^{13}C\{^1H\}$ resonances corresponding to **3** were omitted from the main text and are labelled with red in the above spectrum. Peak at 29.85 ppm is due to H-Grease impurity.

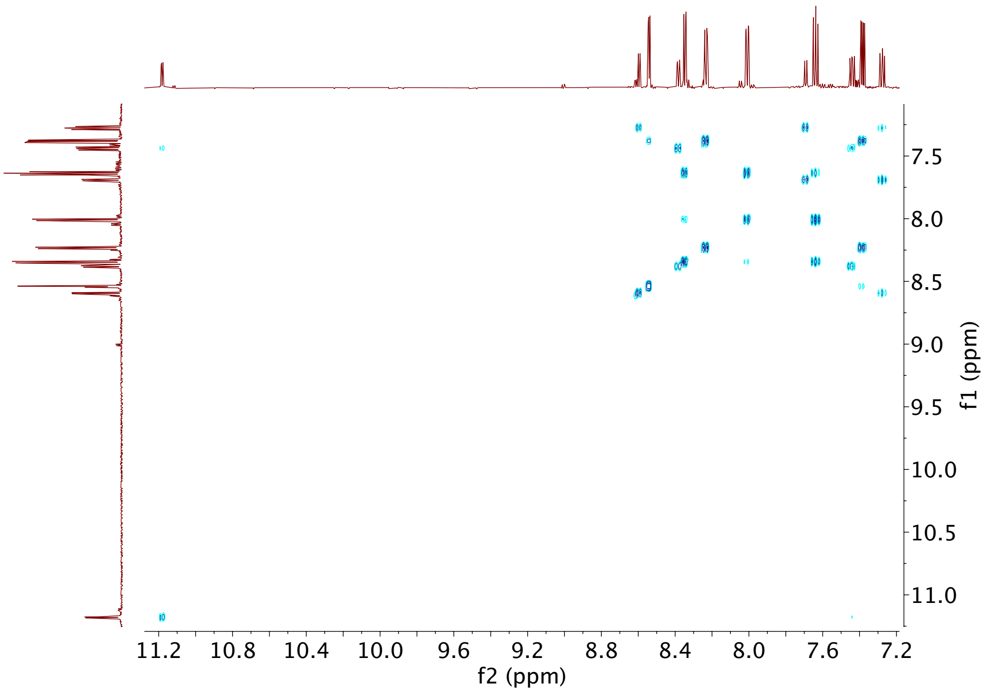

Figure S22. COSY Spectrum of $(SbQ_3)PtCl(OAc)$ (**1a**) (400 MHz, CD_2Cl_2).

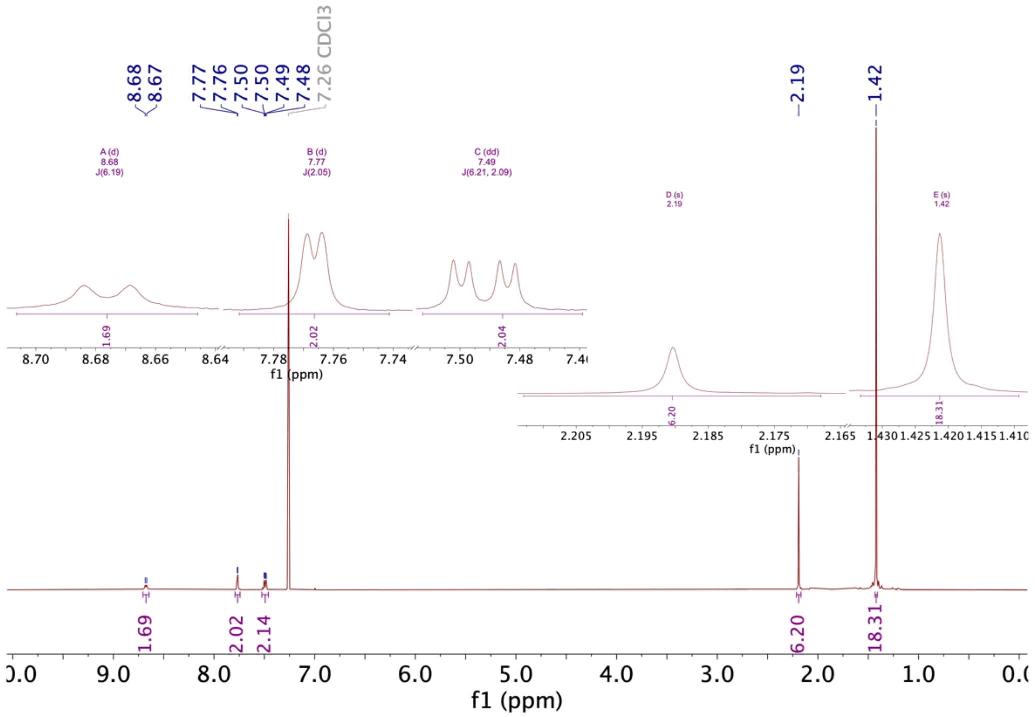

Figure S23. ^1H NMR spectrum of $(^tbpy)Pt(OAc)_2$ (**3**) (600 MHz, $CDCl_3$).

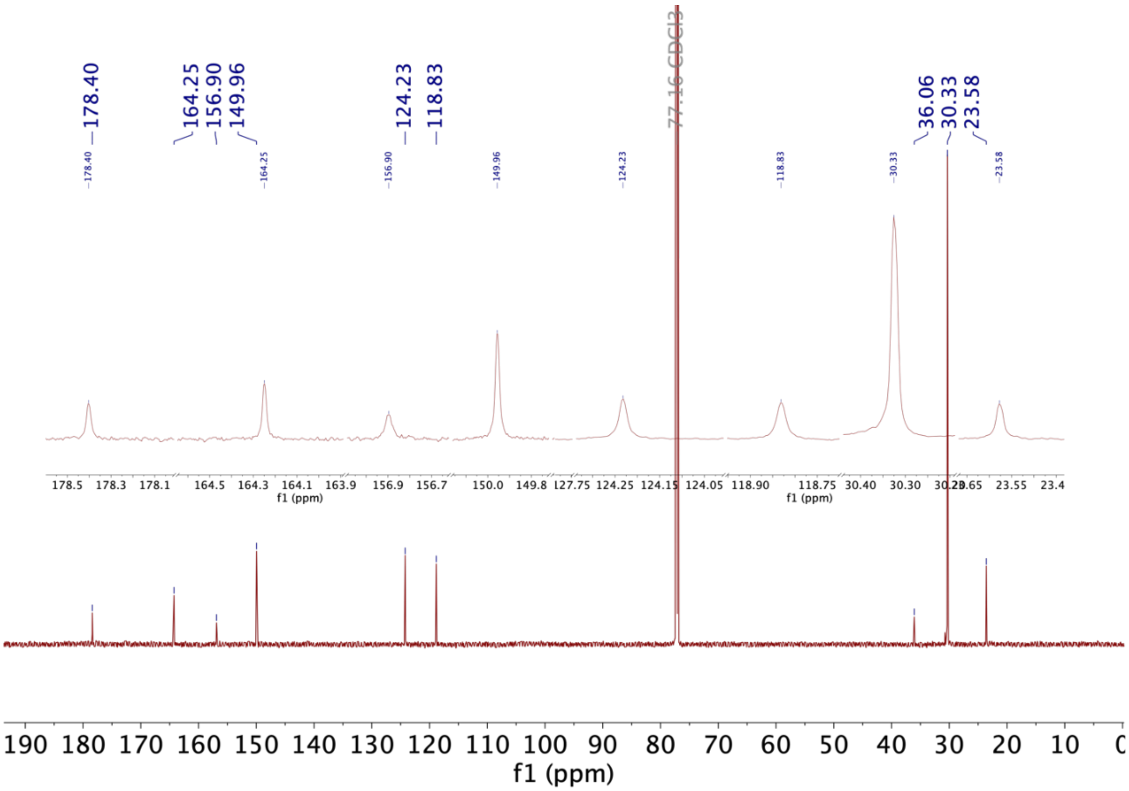

Figure S24. $^{13}C\{^{1}H\}$ NMR spectrum of (^{t}bpy)Pt(OAc)$_2$ (**3**). (201 MHz, $CDCl_3$).

3. X-Ray Crystal Structure Data

Single crystals of **1a** and **3** were coated with Paratone oil and mounted on a MiTeGen MicroLoop. The X-ray intensity data was measured on a Bruker D8 Venture Photon III Kappa four-circle diffractometer system equipped with an Incoatec IμS 3.0 micro-focus sealed X-ray tube (Mo K_α, λ = 0.71073 Å) and a HELIOS double bounce multilayer mirror monochromator. The frames were integrated with the Bruker SAINT software package[1] using a narrow-frame algorithm. Data were corrected for absorption effects using the Multi-Scan method (SADABS).[1] The structures were solved and refined using the Bruker SHELXTL Software Package[2] within APEX5[1] and OLEX2.[3] All the non-hydrogen atoms were refined anisotropically. Hydrogen atoms were placed in geometrically calculated positions with $U_{iso} = 1.2U_{equiv}$ of the parent atom ($U_{iso} = 1.5U_{equiv}$ for methyl).

Table S1. Crystal structure data table for (SbQ$_3$)PtCl(OAc) (**1a**) and (^{t}bpy)Pt(OAc)$_2$ (**3**).

	**1a**	**3**
CCDC number	2351467	2403658
Empirical formula	$C_{32}H_{24}Cl_{10}N_3O_2PtSb$	$C_{45}H_{61}Cl_3N_4O_8Pt_2$
Formula weight	1153.88	1273.55
Temperature [K]	100(2)	100(2)
Wavelength [Å]	0.71073	0.71073
Crystal size [mm^3]	0.076 × 0.166 × 0.187	0.061×0.07×0.165
Crystal habit	yellow plate	yellow prism
Crystal system	triclinic	triclinic
Space group	P -1	P -1
a [Å]	11.2783(4)	12.9936(5)
b [Å]	11.4446(5)	14.2696(7)
c [Å]	15.3008(7)	15.9089(6)
α [°]	98.9190(10)	104.1850(10)
β [°]	93.2500(10)	109.9180(10)
γ [°]	103.2970(10)	108.055(2)
Volume [Å^3]	1889.99(14)	2425.39(18)
Z	2	2
ρ_{calc} [gcm^{-3}]	2.028	1.744
μ [mm^{-1}]	5.154	5.968
F(000)	1104	1251
θ range [°]	1.86 to 25.72	2.26 to 28.46
Index ranges	$-13 \le h \le 12$<br>$-13 \le k \le 13$<br>$-18 \le l \le 18$	$-17 \le h \le 17$<br>$-19 \le k \le 19$<br>$-21 \le l \le 17$
Reflections collected	27478	75700
Independent reflections	7133 [R_{int} = 0.0501]	12104 [R_{int} = 0.0488]
Data / Restraints / Parameters	7133 / 0 / 443	12104 / 19 / 636
Goodness-of-fit on F^2	1.029	1.019
Final *R* indexes [$I \ge 2\sigma(I)$]	R_1 = 0.0313<br>wR_2 = 0.0759	R_1 = 0.0301<br>wR_2 = 0.0646
Final *R* indexes [all data]	R_1 = 0.0390<br>wR_2 = 0.0805	R_1 = 0.0429<br>wR_2 = 0.0703
Largest peak/hole [eÅ^{-3}]	2.565/−0.774	1.58/−1.35

4. DFT Comparison of Complex 1 vs. 3, DFT Basis Set Data, Cartesian Coordinates and Absolute Energies for the Calculated Structures

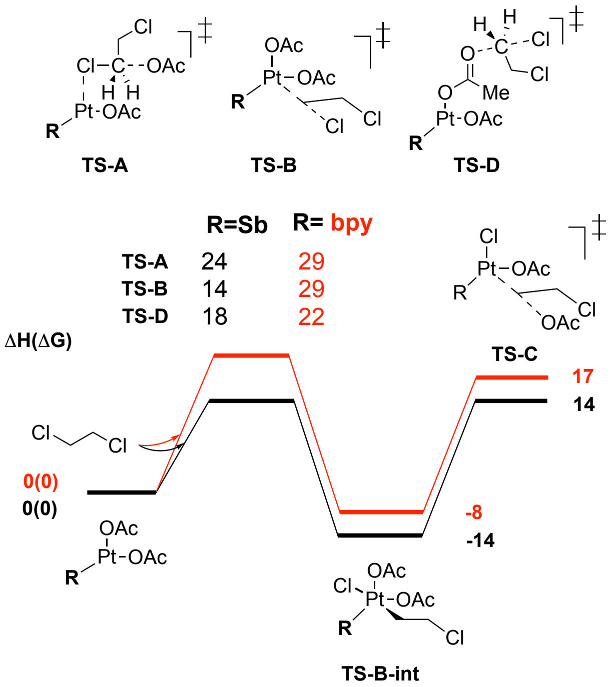

Scheme S1. General mechanisms for the first reaction step of acetoxylation examined with DFT calculations. B2-PLYP/def2TZVPD//M06/def2SVP calculated enthalpies and free energies relative to starting Pt complex. Enthalpies and free energies were calculated at 100 ºC.

Table S2. Effect of basis sets on acetoxylation reaction mechanisms. All the values (ΔH (ΔG) in kcal/mol) are with respect to zero.

Structure/Method	B2PLYP-D3BJ/Def2TZVPD	B2PLYP-D3BJ/Def2TZVPP
**TS-A**	24 (43)	24 (43)
**TS-B**	14 (31)	17 (34)
**TS-B intermediate**	-14 (7)	-12 (9)
**TS-C**	28 (32)	28 (34)
**TS-D**	18 (34)	20 (35)

All the cartesian coordinates optimized at M06/def2-SVP// B2PLYP(D3BJ)/def2-TZVPD level of theory. Energy values are in Hartree. Here, Q=quinoline and Ph=phenyl

1

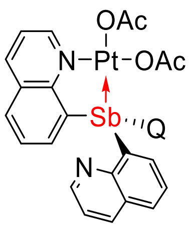

FINAL SINGLE POINT ENERGY = -2019.67495418195

Zero-point correction = 0.479453

Thermal correction to Energy = 0.534351

Thermal correction to Enthalpy = 0.535532

Thermal correction to Gibbs Free Energy = 0.376265

A

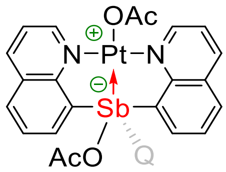

FINAL SINGLE POINT ENERGY = -2019.66126015624

Zero-point correction = 0.479241

Thermal correction to Energy = 0.534074

Thermal correction to Enthalpy = 0.535256

Thermal correction to Gibbs Free Energy = 0.376036

Ion Pair with two quinoline binding to Pt

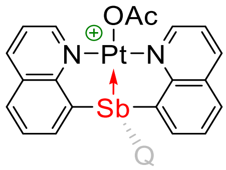

FINAL SINGLE POINT ENERGY = -1791.06612577934

Zero-point correction = 0.429538

Thermal correction to Energy = 0.476319

Thermal correction to Enthalpy = 0.477500

Thermal correction to Gibbs Free Energy = 0.338513

Homolysis of Pt-OAc

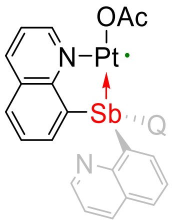

FINAL SINGLE POINT ENERGY = -1791.18165907835

Zero-point correction = 0.426642

Thermal correction to Energy = 0.474519

Thermal correction to Enthalpy = 0.475700

Thermal correction to Gibbs Free Energy = 0.330321

Quinoline ligand slippage

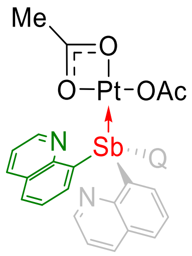

FINAL SINGLE POINT ENERGY = -2019.63718973808

Zero-point correction = 0.478632

Thermal correction to Energy = 0.533921

Thermal correction to Enthalpy = 0.535102

Thermal correction to Gibbs Free Energy = 0.374464

Electron transfer complex

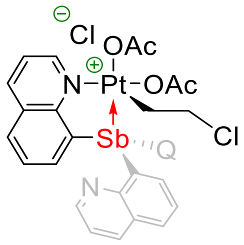

FINAL SINGLE POINT ENERGY = -2479.76385324961

Zero-point correction = 0.480876

Thermal correction to Energy = 0.537244

Thermal correction to Enthalpy = 0.538426

Thermal correction to Gibbs Free Energy = 0.375803

TS-A

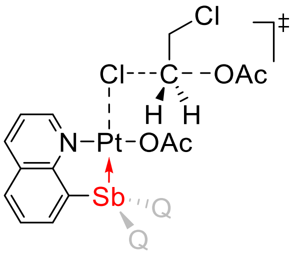

FINAL SINGLE POINT ENERGY = -2019.674954181955

Zero-point correction = 0.537528

Thermal correction to Energy = 0.600314

Thermal correction to Enthalpy = 0.601495

Thermal correction to Gibbs Free Energy = 0.427447

TS-B

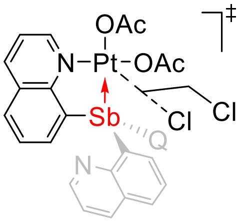

FINAL SINGLE POINT ENERGY = -3018.404992963028

Zero-point correction = 0.536267

Thermal correction to Energy = 0.599635

Thermal correction to Enthalpy = 0.600816

Thermal correction to Gibbs Free Energy = 0.422772

TS-B intermediate

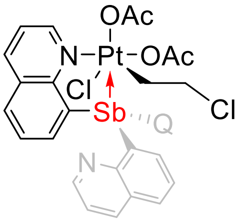

FINAL SINGLE POINT ENERGY = -3018.451012062124

Zero-point correction = 0.538424

Thermal correction to Energy = 0.600411

Thermal correction to Enthalpy = 0.601592

Thermal correction to Gibbs Free Energy = 0.430998

TS-C

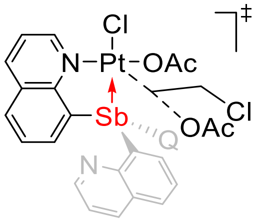

FINAL SINGLE POINT ENERGY = -3018.405256474680

Zero-point correction = 0.536722

Thermal correction to Energy = 0.599787

Thermal correction to Enthalpy = 0.600968

Thermal correction to Gibbs Free Energy = 0.425300

TS-D

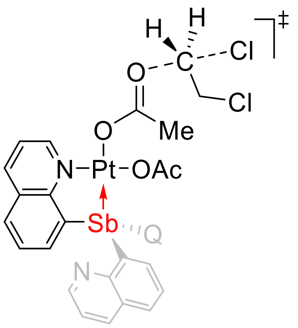

FINAL SINGLE POINT ENERGY = -3018.3990428071

Zero-point correction = 0.537136

Thermal correction to Energy = 0.600379

Thermal correction to Enthalpy = 0.601560

Thermal correction to Gibbs Free Energy = 0.421397

1a

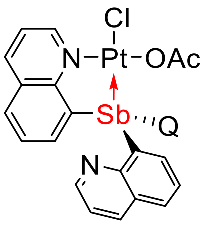

FINAL SINGLE POINT ENERGY = -2251.36169326049

Zero-point correction = 0.429940

Thermal correction to Energy = 0.479507

Thermal correction to Enthalpy = 0.480688

Thermal correction to Gibbs Free Energy = 0.334455

TS-E

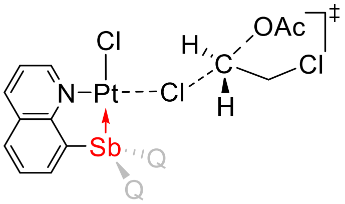

FINAL SINGLE POINT ENERGY = -3250.06616103917

Zero-point correction = 0.486657

Thermal correction to Energy = 0.544899

Thermal correction to Enthalpy = 0.546080

Thermal correction to Gibbs Free Energy = 0.380457

TS-F

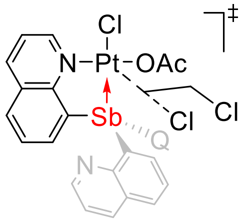

FINAL SINGLE POINT ENERGY = -3250.09077732256

Zero-point correction = 0.486525

Thermal correction to Energy = 0.544864

Thermal correction to Enthalpy = 0.546046

Thermal correction to Gibbs Free Energy = 0.379433

TS-F Intermediate

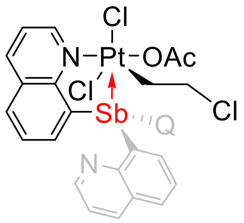

FINAL SINGLE POINT ENERGY = -3250.1391

Zero-point correction = 0.488321

Thermal correction to Energy = 0.546491

Thermal correction to Enthalpy = 0.547672

Thermal correction to Gibbs Free Energy = 0.383942

TS-G

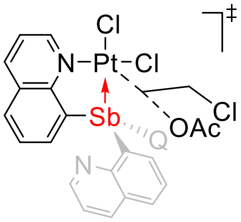

FINAL SINGLE POINT ENERGY = -3250.0798187136

Zero-point correction = 0.485922

Thermal correction to Energy = 0.544546

Thermal correction to Enthalpy = 0.545728

Thermal correction to Gibbs Free Energy = 0.377495

TS-H

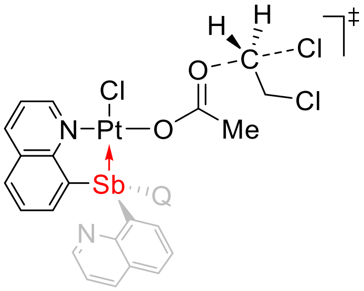

FINAL SINGLE POINT ENERGY = -3250.0775406655

Zero-point correction = 0.487539

Thermal correction to Energy = 0.545399

Thermal correction to Enthalpy = 0.546580

Thermal correction to Gibbs Free Energy = 0.381001

1b

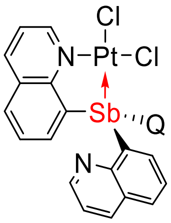

FINAL SINGLE POINT ENERGY = -2483.03934308861

Zero-point correction = 0.379775

Thermal correction to Energy = 0.424505

Thermal correction to Enthalpy = 0.425686

Thermal correction to Gibbs Free Energy = 0.289313

2

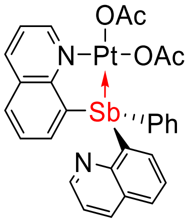

FINAL SINGLE POINT ENERGY = -1850.05177158398

Zero-point correction = 0.444218

Thermal correction to Energy = 0.495268

Thermal correction to Enthalpy = 0.496449

Thermal correction to Gibbs Free Energy = 0.344253

TS-A$_{(Ph)}$

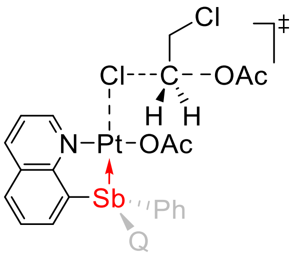

FINAL SINGLE POINT ENERGY = -2848.76752744412

Zero-point correction = 0.502412

Thermal correction to Energy = 0.561059

Thermal correction to Enthalpy = 0.562240

Thermal correction to Gibbs Free Energy = 0.398113

TS-B(Ph)

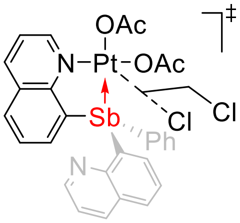

FINAL SINGLE POINT ENERGY = -2848.77819789995

Zero-point correction = 0.500988

Thermal correction to Energy = 0.560441

Thermal correction to Enthalpy = 0.561622

Thermal correction to Gibbs Free Energy = 0.392165

TS-B(Ph) Intermediate

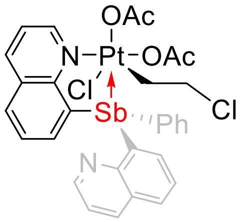

FINAL SINGLE POINT ENERGY = -2848.83647967961

Zero-point correction = 0.503370

Thermal correction to Energy = 0.562524

Thermal correction to Enthalpy = 0.563705

Thermal correction to Gibbs Free Energy = 0.397336

TS-C(Ph)

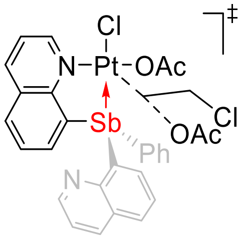

FINAL SINGLE POINT ENERGY = -2848.77686547652

Zero-point correction = 0.501032

Thermal correction to Energy = 0.560386

Thermal correction to Enthalpy = 0.561567

Thermal correction to Gibbs Free Energy = 0.392169

TS-D(Ph)

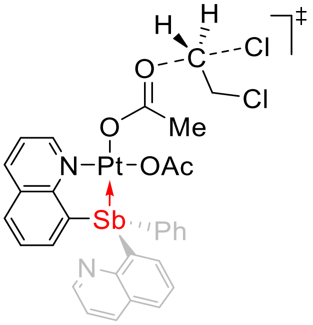

FINAL SINGLE POINT ENERGY = -2848.77509171826

Zero-point correction = 0.502009

Thermal correction to Energy = 0.561348

Thermal correction to Enthalpy = 0.562529

Thermal correction to Gibbs Free Energy = 0.390853

2a

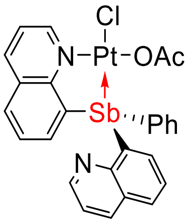

FINAL SINGLE POINT ENERGY = -2081.73883770556

Zero-point correction = 0.394502

Thermal correction to Energy = 0.440220

Thermal correction to Enthalpy = 0.441401

Thermal correction to Gibbs Free Energy = 0.302729

TS-E$_{(Ph)}$

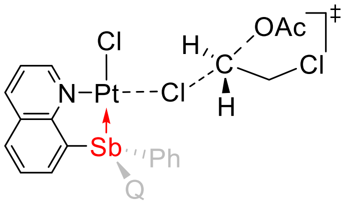

FINAL SINGLE POINT ENERGY = -3080.44112428195

Zero-point correction = 0.451155

Thermal correction to Energy = 0.505715

Thermal correction to Enthalpy = 0.506896

Thermal correction to Gibbs Free Energy = 0.347743

TS-F$_{(Ph)}$

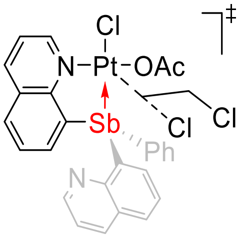

FINAL SINGLE POINT ENERGY = -3080.46480645048

Zero-point correction = 0.450942

Thermal correction to Energy = 0.505423

Thermal correction to Enthalpy = 0.506604

Thermal correction to Gibbs Free Energy = 0.348460

TS-F$_{(Ph)}$ Intermediate

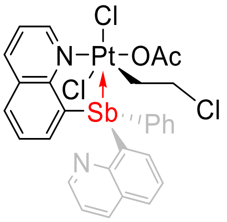

FINAL SINGLE POINT ENERGY = -3080.51985694401

Zero-point correction = 0.452987

Thermal correction to Energy = 0.507374

Thermal correction to Enthalpy = 0.508555

Thermal correction to Gibbs Free Energy = 0.351773

TS-G$_{(Ph)}$

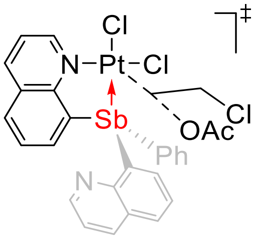

FINAL SINGLE POINT ENERGY = -3080.45124425586

Zero-point correction = 0.450266

Thermal correction to Energy = 0.505106

Thermal correction to Enthalpy = 0.506288

Thermal correction to Gibbs Free Energy = 0.345669

TS-H$_{(Ph)}$

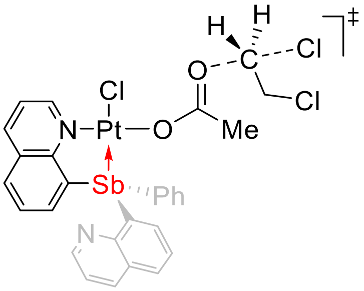

FINAL SINGLE POINT ENERGY = -3080.45111317668

Zero-point correction = 0.452207

Thermal correction to Energy = 0.506226

Thermal correction to Enthalpy = 0.507407

Thermal correction to Gibbs Free Energy = 0.349228

2b

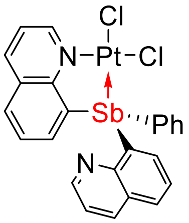

FINAL SINGLE POINT ENERGY = -2313.41489831339

Zero-point correction = 0.344407

Thermal correction to Energy = 0.385238

Thermal correction to Enthalpy = 0.386419

Thermal correction to Gibbs Free Energy = 0.258640

3

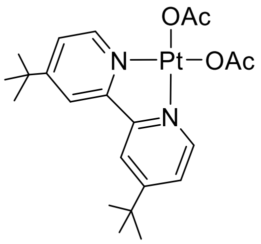

FINAL SINGLE POINT ENERGY = -1385.65045926934

Zero-point correction = 0.483634

Thermal correction to Energy = 0.531506

Thermal correction to Enthalpy = 0.532687

Thermal correction to Gibbs Free Energy = 0.389787

TS-A$_{(t\text{-}bpy)}$

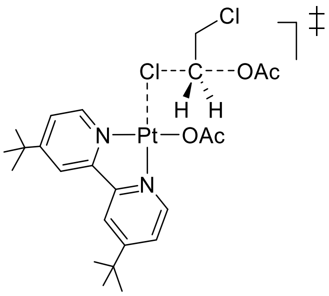

FINAL SINGLE POINT ENERGY = -2384.35697269648

Zero-point correction = 0.540919

Thermal correction to Energy = 0.595899

Thermal correction to Enthalpy = 0.597080

Thermal correction to Gibbs Free Energy = 0.440078

TS-B$_{(t\text{-}bpy)}$

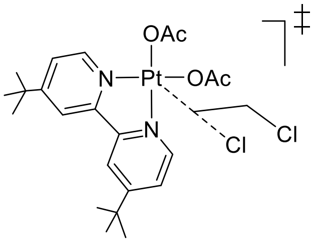

FINAL SINGLE POINT ENERGY = -2384.35699981934

Zero-point correction = 0.541437

Thermal correction to Energy = 0.597124

Thermal correction to Enthalpy = 0.598305

Thermal correction to Gibbs Free Energy = 0.439864

TS-B-int$_{(t\text{-}bpy)}$

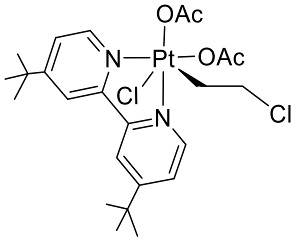

FINAL SINGLE POINT ENERGY = -2384.41796128209

Zero-point correction = 0.542781

Thermal correction to Energy = 0.598784

Thermal correction to Enthalpy = 0.599966

Thermal correction to Gibbs Free Energy = 0.442424

TS-C$_{(t\text{-}bpy)}$

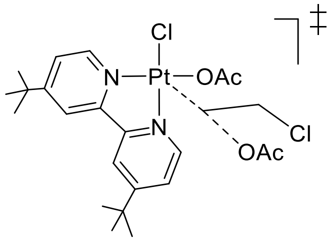

FINAL SINGLE POINT ENERGY = -2384.37682180695

Zero-point correction = 0.540524

Thermal correction to Energy = 0.596846

Thermal correction to Enthalpy = 0.598027

Thermal correction to Gibbs Free Energy = 0.436002

TS-D$_{(t\text{-}bpy)}$

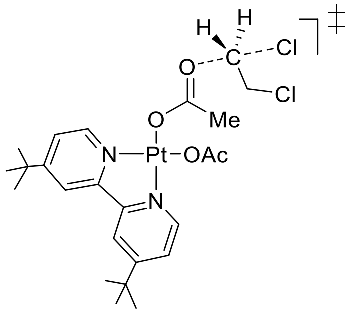

FINAL SINGLE POINT ENERGY = -2384.3695705377

Zero-point correction = 0.542284

Thermal correction to Energy = 0.597842

Thermal correction to Enthalpy = 0.599023

Thermal correction to Gibbs Free Energy = 0.439678

5. References

1. Bruker *Saint; SADABS; APEX3.*, Bruker AXS Inc.: Madison, Wisconsin, USA., 2012.

2. Sheldrick, G. M., SHELXT - integrated space-group and crystal-structure determination. *Acta Crystallogr A Found Adv* **2015,** *A71*, 3-8. doi: 10.1107/s2053273314026370

3. Dolomanov, O.; Bourhis, L.; Gildea, R.; Howard, J.; Puschmann, H., OLEX2: A complete structure solution, refinement and analysis program. *J. Appl. Cryst.* **2009,** *42*, 339-341. doi: 10.1107/S0021889808042726
